# Diagnostic performance of artificial intelligence in detecting oral potentially malignant disorders and oral cancer using medical diagnostic imaging: a systematic review and meta-analysis

**DOI:** 10.3389/froh.2024.1494867

**Published:** 2024-11-06

**Authors:** Rakesh Kumar Sahoo, Krushna Chandra Sahoo, Girish Chandra Dash, Gunjan Kumar, Santos Kumar Baliarsingh, Bhuputra Panda, Sanghamitra Pati

**Affiliations:** ^1^ School of Public Health, Kalinga Institute of Industrial Technology (KIIT) Deemed to be University, Bhubaneswar, India; ^2^ Health Technology Assessment in India (HTAIn), ICMR-Regional Medical Research Centre, Bhubaneswar, India; ^3^ Health Technology Assessment in India (HTAIn), Department of Health Research, Ministry of Health & Family Welfare, Govt. of India, New Delhi, India; ^4^ All India Institute of Medical Sciences, Jodhpur, India; ^5^ Kalinga Institute of Dental Sciences, KIIT Deemed to be University, Bhubaneswar, India; ^6^ School of Computer Engineering, KIIT Deemed to be Uuniversity, Bhubaneswar, India

**Keywords:** oral cancer, AI algorithms, diagnostic performance, deep learning, early detection, medical diagnostic imaging, OPMDs

## Abstract

**Objective:**

Oral cancer is a widespread global health problem characterised by high mortality rates, wherein early detection is critical for better survival outcomes and quality of life. While visual examination is the primary method for detecting oral cancer, it may not be practical in remote areas. AI algorithms have shown some promise in detecting cancer from medical images, but their effectiveness in oral cancer detection remains Naïve. This systematic review aims to provide an extensive assessment of the existing evidence about the diagnostic accuracy of AI-driven approaches for detecting oral potentially malignant disorders (OPMDs) and oral cancer using medical diagnostic imaging.

**Methods:**

Adhering to PRISMA guidelines, the review scrutinised literature from PubMed, Scopus, and IEEE databases, with a specific focus on evaluating the performance of AI architectures across diverse imaging modalities for the detection of these conditions.

**Results:**

The performance of AI models, measured by sensitivity and specificity, was assessed using a hierarchical summary receiver operating characteristic (SROC) curve, with heterogeneity quantified through I^2^ statistic. To account for inter-study variability, a random effects model was utilized. We screened 296 articles, included 55 studies for qualitative synthesis, and selected 18 studies for meta-analysis. Studies evaluating the diagnostic efficacy of AI-based methods reveal a high sensitivity of 0.87 and specificity of 0.81. The diagnostic odds ratio (DOR) of 131.63 indicates a high likelihood of accurate diagnosis of oral cancer and OPMDs. The SROC curve (AUC) of 0.9758 indicates the exceptional diagnostic performance of such models. The research showed that deep learning (DL) architectures, especially CNNs (convolutional neural networks), were the best at finding OPMDs and oral cancer. Histopathological images exhibited the greatest sensitivity and specificity in these detections.

**Conclusion:**

These findings suggest that AI algorithms have the potential to function as reliable tools for the early diagnosis of OPMDs and oral cancer, offering significant advantages, particularly in resource-constrained settings.

**Systematic Review Registration:**

https://www.crd.york.ac.uk/, PROSPERO (CRD42023476706).

## 1 Introduction

Cancer is a predominant cause of mortality and a major obstacle to enhancing global survival outcomes. Oral cancer, a critical global health issue, shows significant prevalence, with approximately 377,713 new cases and 177,757 deaths reported annually worldwide (1–3). The projections from the World Health Organisation (WHO) indicate that the rates of incidence and mortality of oral cancer in Asia are expected to rise to 374,000 and 208,000, respectively, by 2040 (4). OSCC (oral squamous cell carcinoma) is the most prevalent form of malignant neoplasm affecting the oral cavity, with low survival rates that vary among ethnicities and age groups. Despite advancements in cancer therapy, mortality rates for oral cancer remain elevated, with an overall 5-year survival rate of approximately 50% (5). Survival rates can reach 65% in high-income countries but drop to as low as 15% in some rural areas, depending on the affected part of the oral cavity (6). Early identification of oral cancer is vital for minimising both morbidity and mortality while optimising patient health and well-being. The diagnosis of pre-malignant and malignant oral cancer generally relies on a comprehensive patient history, thorough clinical examination, and histopathological verification of epithelial changes (7). The World Health Organisation (WHO) classification system stratifies epithelial dysplasia into mild, moderate, or severe categories, determined by the severity of cytological atypia and architectural disruption within the epithelial layer. Clinicians can evaluate the patient's prognosis and devise an appropriate treatment plan by correlating clinical observations with histological findings. Histopathological analysis remains the definitive standard for diagnosing oral potentially malignant disorders (OPMDs) (8). Currently, visual examination by a trained clinician is the primary detection method, but it is subject to variability due to lighting conditions and clinician expertise, which can reduce accuracy (9). In resource-limited environments, the scarcity of trained specialists and healthcare services impedes timely diagnosis and diminishes survival rates. Conventional oral examinations and biopsies, while gold standards, are not appropriate for screening in these areas (4). There is growing interest in using artificial intelligence (AI) models for the early screening of oral cancer in under-resourced and remote areas to address existing limitations.

AI is a rapidly evolving technology that helps with big data analysis, decision-making, and simulation of human thought processes (10). Deep learning, a subfield of AI, is concerned with convolutional neural networks (CNNs) that learn from large datasets and make accurate predictions, particularly in image classification and medical image analysis tasks (11, 12). Recent improvements in deep learning (DL) algorithms have shown that they are very good at finding cancerous lesions in medical imaging methods, such as CT scans for finding lung cancer and mammograms for checking for breast cancer (13). However, we have yet to fully investigate the potential for automatic detection of oral cancer in images. Disease detection through photographic and histopathological medical images is a crucial aspect of contemporary diagnostic medicine. Photographic imaging techniques, such as MRI (magnetic resonance imaging), CT (computed tomography), and x-rays, mobile captured lesions images enable non-invasive visualisation of internal structures, aiding in the detection and characterisation of various conditions (14).

Advancements in artificial intelligence applications further contribute to the analysis of these images, aiding in faster and more accurate disease detection. This multidimensional approach improves diagnostic precision, which leads to better treatment planning and patient outcomes. At present, there is an absence of a thorough quantitative assessment of the evidence regarding AI-based techniques for detecting oral cancer and OPMDs. This research intends to conduct a comprehensive review and meta-analysis of existing studies evaluating the effectiveness of AI algorithms for identifying both oral cancer and OPMDs.

## 2 Materials and methods

The systematic review was registered with the International Prospective Register of Systematic Reviews (PROSPERO), under Registration Number: CRD42023476706 (https://www.crd.york.ac.uk/prospero/display_record.php?RecordID=476706). The review adhered to the Preferred Reporting Items for Systematic Reviews and Meta-Analysis (PRISMA) guidelines.

### 2.1 Databases & search strategy

We conducted an extensive search of the literature to identify all relevant studies by systematically querying the electronic databases PubMed, Scopus, and IEEE. We included articles published in English up to December 31, 2023. The detailed search strategy related to the keywords and concepts “Machine Learning (ML),” “Deep Learning (DL),” “Artificial Intelligence (AI),” “Oral Cancer,” “Oral Pre-cancer,” “Oral Lesions,” and “Diagnostic Medical Images.” We combined each concept's MeSH terms and keywords with “OR” and then joined the concepts with the “AND” Boolean operator. Specific search strategies were tailored for each database (Supplementary File S1).

Two separate reviewers conducted the study screening based on established eligibility criteria, with the literature being organised using EndNote X9.3.3 (Clarivate Analytics, London, UK). Repeated or non-relevant studies were excluded from consideration. In the initial screening phase, the reviewers evaluated the titles and abstracts of articles, classifying them as relevant, irrelevant, or uncertain. Articles considered irrelevant by both reviewers were removed, while those classified as uncertain underwent further review by a third reviewer. During the secondary screening, potentially eligible articles identified from the initial review were assessed by two separate reviewers based on the eligibility criteria. Any disagreements during the full-text review were resolved by involving a third additional reviewer.

### 2.2 Eligibility criteria

This study includes original research articles focused on the use of AI technologies for diagnosing OPMDs and oral cancer through medical imaging. The included studies provide performance metrics such as sensitivity, specificity, and accuracy, or provide detailed data from the 2 × 2 confusion matrix, covering TP (true positives), TN (true negatives), FP (false positives), and FN (false negatives). Research articles were excluded based on the following criteria: repetition, irrelevant types (including preclinical studies, individual case reports, review articles, or conference proceedings), insufficient data, or lack of reporting on the specified outcomes. These standards were implemented to ensure the rigour and validity of the selected research while minimising potential biases and inaccuracies.

### 2.3 Data extraction and quality assessment

Two separate reviewers carried out the data extraction process, and any discrepancies were resolved by consulting a third additional reviewer. Data were retrieved using a predefined, pre-tested data extraction sheet designed for this study. The sheet included detailed information on author details, year of publication, image types, machine learning and deep learning models, country, TP, TN, FP, FN, sensitivity, accuracy, specificity, continent, World Bank income groups, WHO region, source of collected dataset, and dataset link. Any discrepancies in data retrieval were addressed through consensus among the entire research team.

In instances of missing or incomplete data, the lead authors of the included studies were reached out to via email. The quality of the included studies was assessed using the Quality Assessment of Diagnostic Accuracy Studies-AI (QUADAS-AI) criteria (15), with evaluations conducted by two independent reviewers. This guideline addresses the risk of bias through four domains: patient selection, index test, reference standard, flow and timing, and applicability concerns through three domains: patient selection, index test, and reference standard. The quality of the methodology employed in the included studies was evaluated using the QUADAS-AI tool in Microsoft Excel (Student—version 365, USA).

### 2.4 Statistical analysis

The performance of the AI models was assessed through a hierarchical summary receiver-operating characteristic (SROC) curve, which generated combined curves with 95% confidence intervals focused on average sensitivity (SE), specificity (SP), diagnostic odds ratio (DOR), and area under the curve (AUC) estimates. When several AI architectures were evaluated within a single study, the system demonstrating the greatest accuracy or the most comprehensive 2 × 2 confusion matrix was incorporated into the overall meta-analysis.

To enhance the robustness of the results, both positive and negative likelihood ratios (LR + and LR-) were calculated, providing valuable insights into the test's capacity to confirm or exclude a diagnosis across different clinical scenarios and translating its diagnostic performance into practical clinical decision-making. Heterogeneity among the studies was evaluated with the I^2^ statistic, followed by subgroup analyses to pinpoint the sources of variability. The subgroup analyses included five categories: (1) different AI models (e.g., CNN, VGG, FCN, ResNet, proposed hybrid models, and others); (2) various image types (e.g., histopathological images, photographic images, and optical coherence tomography); (3) diagnostic categories (oral cancer, OPMD, and both); (4) country income levels (high-income wise, upper-middle income and lower-middle income wise); and 5) WHO regions (Americas, Eastern Mediterranean, South-East Asia). All statistical meta-analyses were conducted using MetaDisc (version 1.4, Spain) with a two-tailed significance level of 0.05 (*α* = 0.05). A cross-hairs plot was constructed using Python (V.3.8.18, Netherlands) to present the discrepancies between sensitivity and specificity estimates (16).

## 3 Results

**Figure 1:**
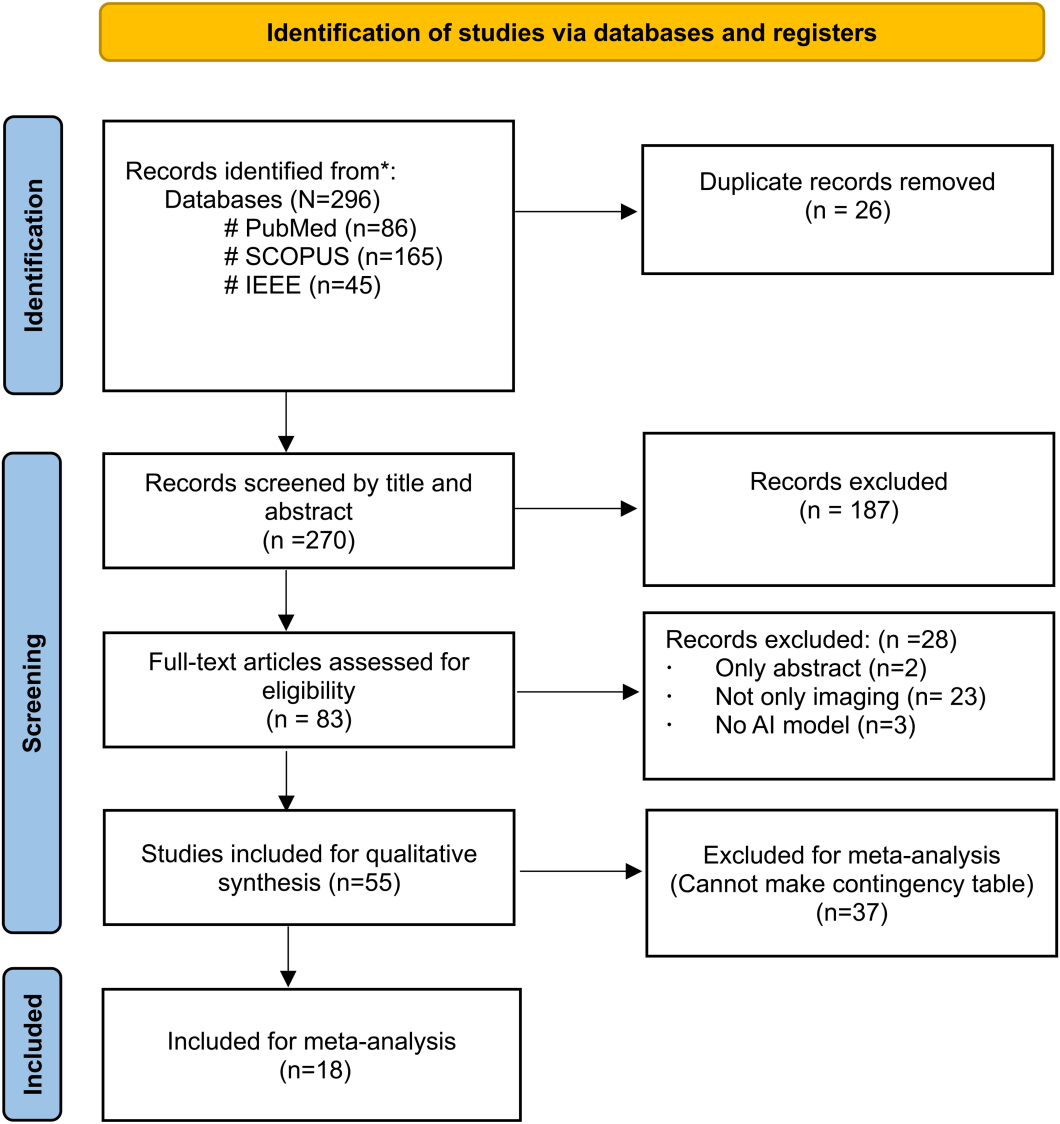
PRISMA flow diagram.

Figure 1 depicts the PRISMA flow diagram for a detailed search and selection of relevant studies. The initial search identified 296 articles. After removing duplicates, 270 were chosen for primary screening. Out of these, 83 were suitable for full-text assessment. The final review included 55 studies (4, 6, 17–69), with only 18 studies considered for the meta-analysis (6, 19–22, 26–31, 36, 44–48, 52–56, 65, 66).

### 3.1 Characteristics of the included studies

**Figure 2:**
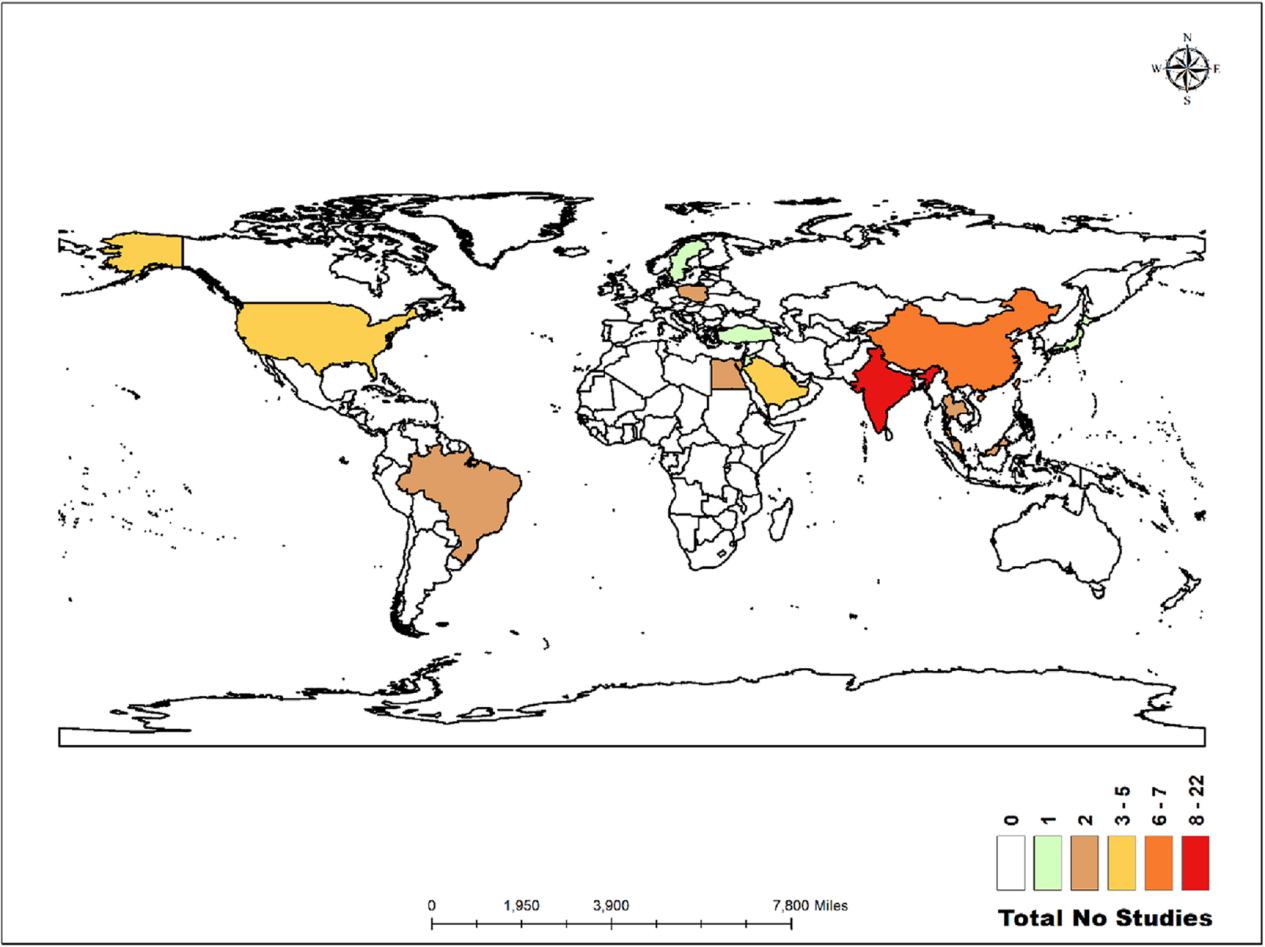
Distribution of the studies across the globe.

The world map in Figure 2 illustrates the distribution of the studies analysed in this review. Twenty-two studies were conducted in India (4, 17, 22, 23, 25, 28, 29, 31, 32, 39, 48, 51, 53, 55–59, 61–63, 67). Similarly, there were seven studies in China (6, 30, 42–44, 50, 52), five in the United States (24, 40, 47, 65, 69), five in Saudi Arabia (19, 20, 27, 36, 37), and two each in Malaysia (21, 54), Thailand (41, 46), Taiwan (60, 66), Egypt (18, 26), Brazil (45, 68), and Poland (34, 35). Additionally, there was one study each in Japan (38), Jordan (33), Türkiye (49), and Sweden (64).

Out of 55 studies, 29 utilised offline patient data from outpatient clinics and inpatient settings across various hospital databases, while 25 relied on online databases. One study incorporated data from both online and offline sources. The studies employed various diagnostic imaging modalities, including photographic images (*n* = 25), histopathological images (*n* = 17), optical coherence tomography (OCT) images (*n* = 4), autofluorescence images (*n* = 4), hyperspectral images (*n* = 2), and one study each used pap smear images, microscopy tissue images, and computed tomography. A total of 42 studies utilised deep learning (DL) models, while one study employed a machine learning (ML) model. Eight studies integrated both DL and ML hybrid techniques for feature extraction and classification. Additionally, two studies developed and proposed their hybrid models, and another two studies also proposed their own hybrid models, comparing their performance with pre-trained DL models for classification. The proposed hybrid models include CADOC-SFOFC, IDL-OSCDC, AIDTL-OCCM, and PSOBER-DBM which blend machine learning and deep learning techniques to enhance predictive model, pattern recognition, and classification. Meanwhile, the studies also explored various DL architectures for image classification, such as CNNs and specialized models like ANN, VGG, ResNet, Fully Convolutional Networks (FCNs), etc. Detailed characteristics of these studies are provided in Table 1.

**Table 1:** Study characteristics.

Authors, Year	country	Income groups	Type of Images	AI Algorithm architecture	DL & ML	Total images	Data Available (TP, TN, FP, FN)	Data sources (online/offline)
Afify et al. 2023 (18)	Egypt	Lower-Middle-Income	Histopathological Images	ResNet-101, GoogLeNet, SqueezeNet, ShuffleNet, AlexNet, DenseNet-201, InceptionResNetV2, EfficientNet-B0, VGG-19, and NASNetMobile	DL	1224	No	Online
Al Duhayyim et al. 2023 (19)	Saudi Arabia	High-Income	Photographic Images	CADOC-SFOFC (Sailfish Optimization and Fusion-Based Classification Model)	Hybrid Model	131	Yes	Online
Alanazi et al. 2022 (20)	Saudi Arabia	High-Income	Photographic Images	Intelligent Deep Learning-Enabled Detection and Classification of Oral Squamous Cell Carcinoma (IDL-OSCDC)	Hybrid Model	131	Yes	Online
Ananthakrishnan et al. 2023 (17)	India	Lower-Middle-Income	Microscopy tissues Images	(ResNet50, ResNet101, ResNet152, ResNet50V2, ResNet101V2, ResNet152V2, Xception, VGG16, VGG19, InceptionV3, InceptionResNetV2, DenseNet201, DenseNet121, DenseNet169) + Random Forest	DL & ML	1,224	No	Offline
Awais et al. 2020 (21)	Malaysia	Upper-Middle-Income	Auto-fluorescence images	K-Nearest Neighbors (KNN)	ML	30	No	Offline
Bansal et al. 2023 (22)	India	Lower-Middle-Income	Photographic Images	CNN	DL	131	Yes	Online
Bhupathia, T.S.C.U.S.R. et al. 2023 (23)	India	Lower-Middle-Income	Photographic Images	CNN	DL	NM	No	Online
Camalan et al. 2021 (24)	USA	High-Income	Photographic Images	ResNetV2	DL	239	No	Online
Das Madhusmita et al. 2023 (25)	India	Lower-Middle-Income	Histopathological Images	VGG16, VGG19, AlexNet, ResNet50, ResNet101, InceptionNet, MobileNet, Proposed 10-Layer CNN	DL	1,224	No	Online
Deif et al. 2022 (26)	Egypt	Lower-Middle-Income	Histopathological Images	XGBoost Classifier, Random Forest Classifier, ANN	DL & ML	1,224	Yes	Online
Fati et al. 2022 (27)	Saudi Arabia	High-Income	Histopathological Images	ANN + (AlexNet, DWT, LBP, FCH, and GLCM), ANN + (ResNet-18, DWT, LBP, FCH, and GLCM), AlexNet + SVM, ResNet-18 + SVM, AlexNet + ANN, ResNet-18 + ANN	DL & ML	5,192	Yes	Online
Figueroa et al. 2022 (28)	India	Lower-Middle-Income	Photographic Images	CNN + GAIN (Guided attention inference network)	DL	NM	No	Offline
Gupta et al. 2020 (29)	India	Lower-Middle-Income	Histopathological Images	CNN	DL	2,557	Yes	Offline
Huang et al.2023 (30)	China	Upper-Middle-Income	Photographic Images	CNN	DL	131	No	Online
James et al. 2021 (31)	India	Lower-Middle-Income	Optical coherence tomography	DenseNet-201 + SVM, Inception-ResNet-v2 + SVM	DL & ML	347	Yes	Offline
Jeyaraj et al. 2019 (32)	India	Lower-Middle-Income	hyperspectral image	CNN	DL	1,300	No	Online
Jubair et al. 2022 (33)	Jordan	Lower middle income	Photographic Images	EfficientNet-B0, VGG19, ResNet101	DL	716	No	Offline
Jurczyszyn et al. 2020 (34)	Poland	High-Income	Photographic Images	ANN	DL	35	No	Offline
Jurczyszyn et al. 2019 (35)	Poland	High-Income	Photographic Images	ANN	DL	63	No	Offline
Lin et al. 2021 (6)	China	Upper-Middle-Income	Photographic Images	VGG16, ResNet50, DenseNet169, HRNet-W18	DL	1,448	Yes	Offline
Marzouk et al. 2022 (36)	Saudi Arabia	High-Income	Photographic Images	AIDTL-OCCM, ResNet-152, Ensemble model, DenseNet-161, Inception-v4, EfficientNet-b4	DL, ML & Proposed hybrid model	131	Yes	Online
Myriam et al. 2023 (37)	Saudi Arabia	High-Income	Photographic Images	PSOBER-DBM, DBN, SVM-Linear, SVM-Gaussian, SVM-Cubic, KNN, Linear Discriminant, Decision Tree	DL, ML & Proposed hybrid model	131	No	Online
Oya et al. 2023 (38)	Japan	High-Income	Histopathological Images	EfficientNet	DL	90	No	Offline
Song,Bofan et al. 2021 (39)	India	Lower-Middle-Income	Photographic Images	VGG19	DL	3,851	No	Offline
Song et al. 2021 (4)	India	Lower-Middle-Income	Auto-fluorescence images	MobileNet	DL	5,025	No	Offline
Uthoff et al. 2018 (40)	USA	High-Income	Auto-fluorescence images	VGG-M	DL	170	No	Offline
Warin et al. 2021 (41)	Thailand	Upper-Middle-Income	Photographic Images	DenseNet121, R-CNN	DL	700	No	Offline
Xu, Shipu, et al. 2019 (42)	China	Upper-Middle-Income	Computed Tomography images	2DCNN, 3DCNN	DL	7,000	No	Offline
Yang, Zihan, et al. 2023 (43)	China	Upper-Middle-Income	Optical coherence tomography	LeNet-5, VGG16, ResNet18, LeNet-5 + DT, LeNet-5 + RF, LeNet-5 + SVM, VGG16 + DT, VGG16 + RF, VGG16 + SVM, ResNet18 + DT, ResNet18 + RF, ResNet18 + SVM	DL & ML	13,799	No	Offline
Yuan, Wei, et al. 2022 (44)	China	Upper-Middle-Income	Optical coherence tomography	VGG-16, GoogLeNet, Inception-V3, ResNet-50, GRAN (LARN + MMD), Local Residual Adaptation Network (LRAN)	DL	26,446	yes	Offline
Zhang, Xinyi, et al. 2023 (45)	Brazil	Upper-Middle-Income	Histopathological Images	CNN-Based Oral Mucosa Risk Stratification Model (OMRS)	DL	14,425	No	Online
Warin, K., et al. 2022 (46)	Thailand	Upper-Middle-Income	Photographic Images	DenseNet-121, ResNet-50, Faster R-CNN, YOLOv4	DL	600	Yes	Offline
Liu, Y et al. 2022 (47)	USA	High-Income	Histopathological Images	DeepLabv3+, U-Net++	DL	39,898	No	Online
Panigrahi, Santisudha, et al. 2023 (48)	India	Lower-Middle-Income	Histopathological Images	VGG16, VGG19, InceptionV3, ResNet50, MobileNet, Proposed Baseline CNN	DL	4,000	Yes	Online & Offline
Keser, Gaye, et al. 2023 (49)	Türkiye	Upper-Middle-Income	Photographic Images	Google Inception V3	DL	137	No	Offline
Yuan, Wei, et al. 2023 (50)	China	Upper-Middle-Income	Optical coherence tomography	VGGNet, GoogLeNet, ResNet, Multi-Level Deep Residual Learning (MDRL)	DL	460	No	Offline
Ünsal, Gürkan, et al. 2023 (51)	India	Lower-Middle-Income	Photographic Images	U-Net	DL	510	No	Offline
Yang, S. Y., et al. 2022 (52)	China	Upper-Middle-Income	Histopathological Images	SVM, Linear Discriminant, CNN	DL & ML	2,025	yes	Offline
Muqeet, Mohd Abdul, et al. 2022 (53)	India	Lower-Middle-Income	Photographic Images	VGG19, InceptionNet-V3, Xception	DL	131	yes	Online
Welikala, Roshan Alex, et al. 2020 (54)	Malaysia	Upper-Middle-Income	Photographic Images	ResNet-101, R-CNN	DL	2,155	yes	Offline
Panigrahi, Santisudha et al. 2019 (55)	India	Lower-Middle-Income	Histopathological Images	CNN, DNN, DCNN, Self-proposed Model	DL	1,000	No	Online
Goswami, Mukul, et al. 2021 (56)	India	Lower-Middle-Income	Photographic Images	CNN	DL	598	yes	Offline
Jenifer Blessy et al. 2023 (57)	India	Lower-Middle-Income	Histopathological Images	Neural Network, Radial Basis Function Neural Network, Chebyshev Neural Network	DL	1,224	No	Online
Kavyashree et al. 2022 (58)	India	Lower-Middle-Income	Histopathological Images	CNN, DenseNet-201, DenseNet-121, DenseNet-169	DL	526	No	Online
Manikandan, J. et al. 2023 (59)	India	Lower-Middle-Income	Photographic Images	CLACHE + GLCM + ICNN	DL	510	No	Online
Chan, Chih-Hung, et al. 2019 (60)	Taiwan	High income	Auto-fluorescence images	Texture model	DL	80	No	Offline
Yadav, Ram Kumar et al. 2023 (61)	India	Lower-Middle-Income	Photographic Images	CNN, K-NN, SVM, Naive Bayes, AdaBoost	DL & ML	131	No	Online
Subha et al. 2023 (62)	India	Lower-Middle-Income	Histopathological Images	CNN	DL	5,192	No	Online
Saraswathi, T et al. 2023 (63)	India	Lower-Middle-Income	Histopathological Images	AlexNet	DL	1,000	No	Online
Wetzer, Elisabeth, et al.2020 (64)	Sweden	High income	Pap-smear sample images	Texture Model, ResNet50, VGG	DL	7755	No	Offline
Xue, Zhiyun, et al.2022 (65)	USA	High income	Photographic Images	ResNet, ViT	DL	2,817	yes	Offline
Huang et al. 2022 (66)	Taiwan	High income	Photographic Images	FCN	DL	221	yes	Offline
Jeyaraj et al. 2019 (67)	India	Lower-Middle-Income	Hyperspectral image	SVM, ResNet	DL & ML	2,400	No	Online
Matias Victória et al.2020 (68)	Brazil	Upper-Middle-Income	Histopathological Images	ResNet-34, ResNet-50	DL	1,934	No	Online
Aljuaid, Abeer et al. 2022 (69)	USA	High income	Histopathological Images	Inception-V3, GoogLeNet Inception-V3	DL	448	No	Offline

DL, deep learning, ML, machine learning.

The studies represented a diverse range of settings, with 25 originating from low- and middle-income countries, 14 from upper-middle-income countries, and 16 from high-income countries. In all included studies, retrospective and online data sources provided pre-annotated datasets, whereas datasets collected prospectively were annotated by specialist dentists. Included studies validated their AI models using internal datasets, with one study additionally performing external validation with experts. The studies focused on validating AI algorithms across various imaging modalities, using metrics such as TP, TN, FP, FN, sensitivity, specificity, and AUC.

### 3.2 Quality assessment

The quality assessment of the studies was assessed using the QUADAS-AI tool (Supplementary File S2). The comprehensive assessment results are depicted in a diagram in the Supplementary Figure. A total of 14 studies showed a low risk of bias in patient selection, while 15 studies demonstrated proper flow and timing management. However, eight studies were at high risk of bias in the index test due to insufficient blinding and inconsistencies. For the reference standard, 10 studies were classified as having a low risk of bias, whereas eight studies exhibited varying levels of risk. Applicability concerns were low during in-patient selection (*n* = 16), but higher for the index test (*n* = 7). Many studies demonstrated robustness in multiple domains; however, significant issues were identified in the index test and reference standard, highlighting areas for improvement in future research designs.

### 3.3 Meta-analysis: pooled performance of AI algorithms

The study evaluates diagnostic accuracy across 18 studies, revealing a high sensitivity of 0.87, identifying 87% of true positive cases, and a specificity of 0.81 recognising 81% of true negative cases. The DOR of 131.63 reflects a strong likelihood of accurate diagnosis, while the SROC curve with an AUC of 0.9758 indicates exceptional diagnostic performance, highlighting the nearly perfect accuracy of the models (Supplementary File S3). These results confirm the reliability and robustness of AI algorithms for precise diagnostic applications. The detailed comparative analysis of pooled sensitivity, specificity, and diagnostic odds ratio (DOR), and the likelihood ratio of various AI Models for detecting oral cancer categorised by image type, oral conditions, and WHO regions are detailed in Table 2.

**Table 2:** Comparative analysis of pooled sensitivity, specificity, diagnostic odds ratio (DOR), and likelihood ratio of Various AI models for detecting OPMDs & oral cancer, categorized by image types, oral conditions, income-wise and wHO regions.

	No of studies	Sensitivity			Specificity			Diagnostic odds ratio			LR+	LR-
		Sensitivity	I^2^ value (%)	*P*-value	Specificity	I^2^ value (%)	*P*-value	DOR	I^2^ value (%)	*P*-value		
Overall (Supplementary file 3)	39	0.87 (0.85–0.89)	98.20%	0.0000	0.81 (0.78–0.84)	99.20%	0.0000	131.63 (90.66–191.14)	99.00%	0.0000	9.11 (7.47–11.10)	0.09 (0.07–0.11)
AI model (Supplementary file 4)												
CNN	5	0.95 (0.92–0.97)	17.3	0.3043	0.95 (0.93–0.97)	0	0.4517	313.92 (167.55–588.15)	2.9	0.3903	14.83 (8.14–27.05)	0.06 (0.04–0.09)
VGG	5	0.90 (0.89–0.91)	37.7	0.1699	0.88 (0.85–0.91)	96.1	0.0000	145.03 (53.30–394.63)	77.4	0.0014	11.47 (5.04–26.09)	0.12 (0.11- 0.13)
ResNET	5	0.92 (0.89–0.95)	93.1	0.0000	0.87 (0.84–0.91)	97.3	0.0000	303.62 (62.91–1,465.27)	87.7	0.0000	19.58 (7.29–52.58)	0.06 (0.02–0.23)
Fully convolution Network	5	0.81 (0.76–0.87)	99.2	0.0000	0.72 (0.68–0.77)	99	0.0000	11.94 (9.43–15.12)	97.2	0.0000	2.97 (2.64–3.33)	0.25 (0.19–0.32)
Proposed Hybrid Model	6	0.91 (0.88–0.94)	96.9	0.0000	0.91 (0.88–0.94)	96.7	0.0000	766.08 (75.44–7,779.58)	95.3	0.0000	20.91 (6.76–64.67)	0.03 (0.01- 0.11)
Others	13	0.90 (0.88–0.91)	92.9	0.0000	0.88 (0.86–0.90)	95.9	0.0000	126.25 (81.41–195.79)	94.6	0.0000	8.03 (6.37–10.11)	0.08 (0.06–0.10)
**Type of Images** (Supplementary File S5)												
Histopathological	15	0.97 (0.95–0.99)	81	0.0000	0.95 (0.93–0.98)	86.4	0.0000	460.83 (216.34–981.60)	79.5	0.0000	15.15 (9.07–25.29)	0.04 (0.02–0.06)
Photographic	17	0.82 (0.79–0.85)	97.7	0.0000	0.73 (0.70–0.77)	98.8	0.0000	23.53 (17.54–31.56)	94.7	0.0000	4.02 (3.49–4.63)	0.20 (0.16–0.25)
Optical coherence tomography	7	0.90 (0.89–0.91)	93.4	0.0000	0.88 (0.86–0.90)	97.2	0.0000	63.45 (48.30–83.35)	96.7	0.0000	7.19 (6.12–8.44)	0.11 (0.10–0.13)
**Oral Condition** (Supplementary File S6)												
Oral Cancer	30	0.91 (0.90–0.91)	92.7	0.0000	0.89 (0.87–0.90)	96.2	0.0000	159.76 (121.46–210.15)	92.8	0.0000	9.81 (8.42–11.43)	0.08 (0.07–0.10)
OPMD	3	0.96 (0.92–1.00)	75.9	0.0157	0.93 (0.90–0.96)	7.8	0.3381	347.93 (157.59–768.17)	0	0.5801	13.29 (8.49–20.81)	0.03 (0.01–0.13)
Both	6	0.81 (0.76–0.86)	99.1	0.0000	0.72 (0.68–0.77)	99	0.0000	12.24 (9.70–15.44)	96.6	0.0000	3.04 (2.70–3.42)	0.28 (0.22–0.35)
**Income-wise** (Supplementary File S7)												
Lower-Middle-Income	14	0.95 (0.93–0.97)	67.3	0.0002	0.90 (0.86–0.94)	80.3	0.0000	213.47 (119.33–381.89)	63.2	0.0008	9.55 (6.07–15.01)	0.06 (0.04–0.08)
High-Income	12	0.82 (0.78–0.86)	98.9	0.0000	0.74 (0.69–0.78)	99.3	0.0000	31.53 (22.15–44.88)	97.3	0.0000	4.33 (3.68–5.10)	0.15 (0.12–0.20)
Upper-Middle-Income	13	0.90 (0.89–0.91)	91.9	0.0000	0.88 (0.87–0.89)	96.2	0.0000	78.26 (59.61–102.75)	94.3	0.0000	8.39 (7.16–9.82)	0.12 (0.10–0.14)
**WHO Region wise** (Supplementary File S8)												
Americas Region (AMR)	2	1.00 (0.98–1.00)	0	1.00	1.00 (0.99–1.00)	0	0.5593	42,983.67 (4,722.25–391,253.31)	0	0.8197	223.03 (83.89–592.94)	0.01 (0.00–0.04)
Eastern Mediterranean Region (EMR)	8	0.99 (0.97–1.00)	74	0.0003	0.96 (0.92–1.00)	92.5	0.0000	714.94 (135.73–3,765.72)	89	0.0000	14.11 (4.88–40.84)	0.02 (0.01–0.07)
South-East Asia Region (SEAR)	13	0.94 (0.92–0.96)	60.3	0.0026	0.91 (0.88–0.95)	77.2	0.0000	228.77 (115.69- 452.36)	65.9	0.0004	11.08 (6.53–18.80)	0.07 (0.04–0.10)
WPR	16	0.86 (0.83–0.89)	99	0.0000	0.80 (0.76–0.85)	99.6	0.0000	43.98 (26.78–72.21)	99.6	0.0000	6.21 (4.76–8.11)	0.16(0.12–0.21)

**Figure 3:**
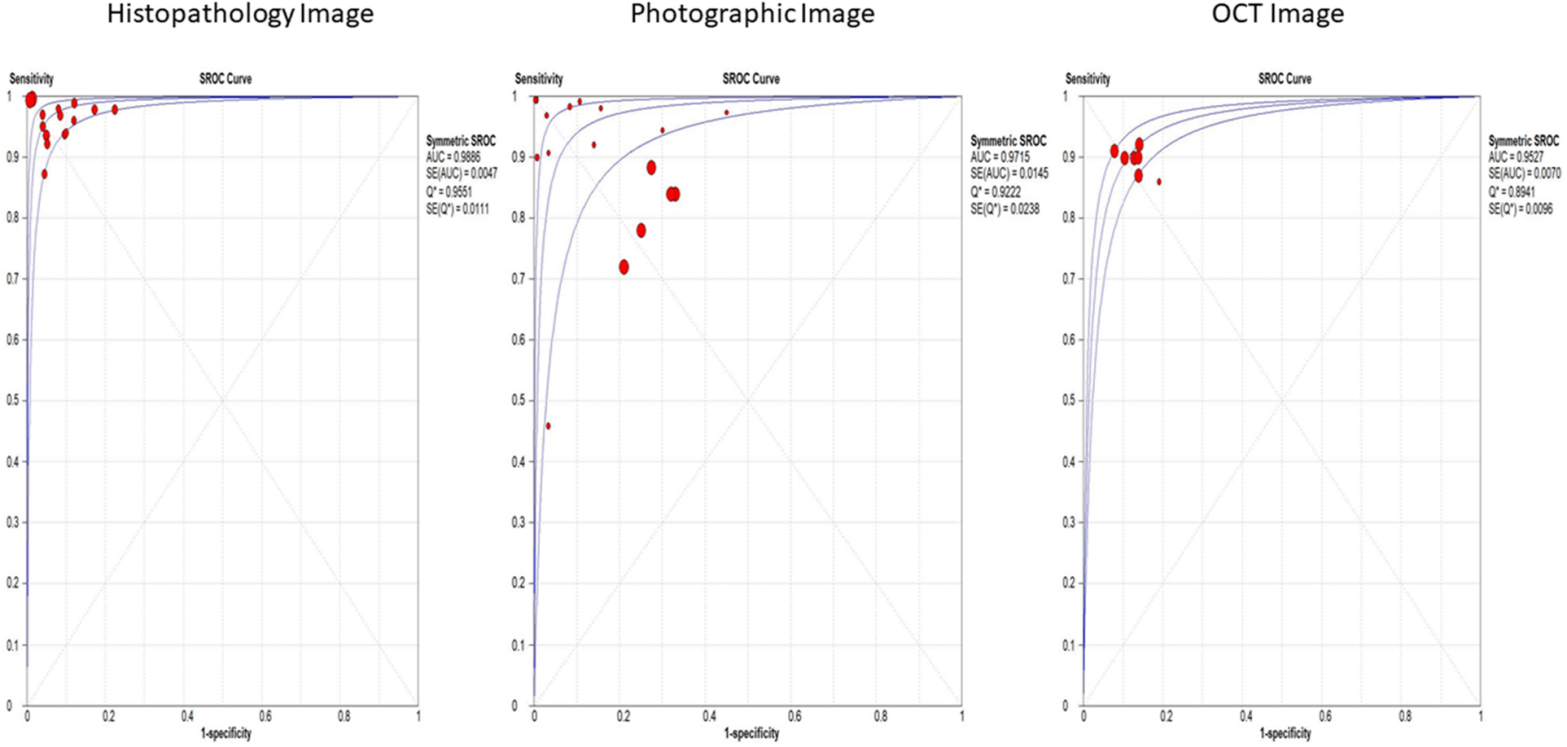
Overall SROC plot for various image-based diagnostic performance of artificial intelligence algorithms for detecting OPMDs & oral cancer.

Histopathological images, evaluated in 15 studies, demonstrated the highest sensitivity and specificity, with values of 97% (95% CI: 95%–99%) and 95% (95% CI: 93%–98%), respectively. These images also had a significantly high diagnostic odds ratio (DOR) of 460.83 (95% CI: 216.34–981.60) and an area under the summary receiver operating characteristic (SROC) curve (AUC) of 0.9886. Photographic images, assessed in 17 studies, showed lower sensitivity and specificity at 82% (95% CI: 79%–85%) and 73% (95% CI: 70%–77%), respectively, with a DOR of 23.53 (95% CI: 17.54–31.56) and an AUC of 0.9715 (Figure 3). Optical coherence tomography, evaluated in 7 studies, had a sensitivity of 90% (95% CI: 89%–91%) and specificity of 88% (95% CI: 86%–90%), with a DOR of 63.45 (95% CI: 48.30–83.35) and an AUC of 0.9527 (Supplementary File S5).

**Figure 4:**
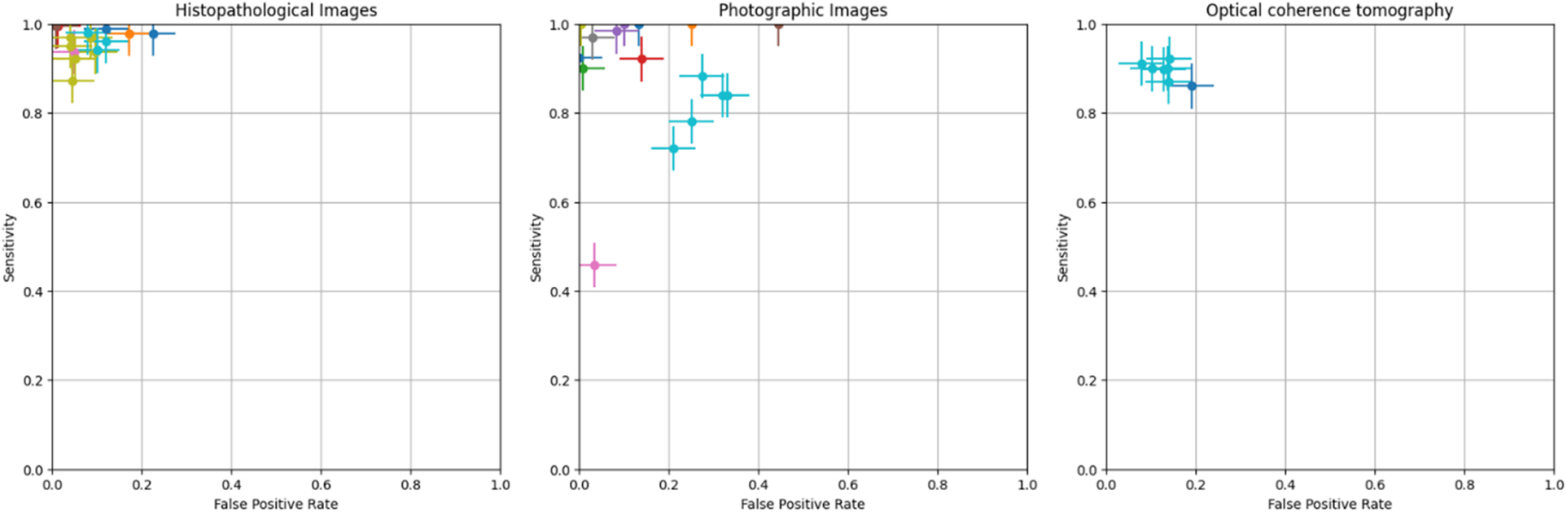
Crosshair plot for various image-based diagnostic performance of artificial intelligence algorithms for detecting OPMDs & oral cancer.

The crosshair plot below depicts the relationship between the FPR (*x*-axis) and sensitivity (*y*-axis) for various data points represented by different colors. It was observed that the majority of the data points were clustered in the top left corner of the plot, indicating high sensitivity (above 0.8) and low FPR (less than 0.3). This suggests that the tested models perform well in terms of accurately identifying TP while generating a low number of FP. A few outliers with lower sensitivity and higher FPR exist, indicating poorer performance in those cases. However, the error bars on each data point show the variability or uncertainty in the measurements (Figure 4).

This study categorised and analysed various AI models used for medical image classification, focusing on their performance. The CNN showed high sensitivity and specificity, with a DOR of 313.92 and an AUC of 0.9846. VGG models exhibited slightly reduced sensitivity and specificity, with a DOR of 145.03 and an AUC of 0.9539. ResNet models demonstrated impressive performance, achieving a sensitivity of 92% and specificity of 87%. Fully convolutional networks had lower performance with a sensitivity of 81% and specificity of 72%. The hybrid AI model for enhanced accuracy showed impressive results, with a sensitivity of 91% and specificity of 91%. Other models, integrating various machine learning techniques and deep learning architectures, demonstrated comparable results. (Supplementary File S4).

Additionally, the study found that oral cancer conditions have a sensitivity of 91% and specificity of 89%, with a diagnostic odds ratio of 159.76 and an AUC of 0.9850. OPMD has a sensitivity of 96% and specificity of 93%, with a DOR of 347.93 and AUC of 0.9849 (Supplementary File S6). In terms of income groups, as classified by the World Bank, diagnostic performance varies: lower-middle-income countries have a sensitivity of 95% and specificity of 90%, while high-income countries exhibit a sensitivity of 82% and specificity of 74%. Upper-middle-income countries show a sensitivity of 90% and a specificity of 88% (Supplementary File S7). Regionally, the Americas Region demonstrated the highest sensitivity and specificity, followed by the Eastern Mediterranean Region, Southeast Asia Region, and Western Pacific Region (Supplementary File S8).

### 3.4 Heterogeneity analysis

A meta-analysis of 18 studies demonstrated that AI models are effective in diagnosing OPMDs and oral cancer using medical diagnostic images, as indicated by a random-effects model analysis. Nevertheless, substantial heterogeneity was observed among the studies, with sensitivity exhibiting an I^2^ of 98.2% and specificity showing an I^2^ of 99.2% (*p* < 0.01). Detailed results from subgroup analyses, which address the potential sources of inter-study variability, are presented in Table 2.

## 4 Discussion

This review presents a comprehensive meta-analysis of AI algorithms in medical imaging, specifically focusing on screening for OPMDs and oral cancer. Majority of studies utilised patient data that was collected offline and employed advanced deep learning architectures, such as CNNs, VGG, ResNET, etc. to analyse visual data. The findings indicate that AI algorithms exhibit a high level of diagnostic accuracy in detecting both oral cancer and OPMDs through medical imaging. The pooled sensitivity and specificity were 87% and 81%, respectively, indicating high diagnostic accuracy. Deep learning algorithms, a subfield of AI, have achieved remarkable success in disease classification through the analysis of various medical images. AI-driven medical diagnostic images have proven to be highly accurate and reliable in detecting tuberculosis, as well as cervical, and breast cancer. In tuberculosis detection, deep learning systems analysing chest x-rays have achieved a sensitivity of more than 95%, significantly reducing radiologists’ workload and enabling timely diagnosis (70). In breast cancer detection, AI models interpreting mammograms have outperformed human experts by reducing both false positives and false negatives (71, 72). Similarly, in cervical cancer, AI-based histopathological image analysis has demonstrated a sensitivity of 91%, highlighting its robustness in disease classification (72). The CNN model demonstrated the highest performance, achieving a sensitivity and specificity of 95% in this review. This was particularly notable when compared to other models such as VGG, ResNet, Inception, etc. which, despite being trained with a large number of parameters and being computationally efficient, did not perform as well. Many studies combine machine learning and deep learning techniques to create hybrid models, which also achieved impressive results, with a sensitivity of more than 95% (17).

AI algorithms demonstrated a sensitivity of 95% and specificity of 90% LIMCs compared to all income groups. This suggests that AI models can be effectively trained and utilised in diverse economic settings, potentially offering higher diagnostic accuracy in LMICs where traditional diagnostic resources are scarce. The implementation of AI-enabled portable devices for screening pre-malignant oral lesions may reduce the disease burden and improve the survival rate of oral cancer patients in LMICs (73). A scoping review by Adeoye, John, et al. highlighted the growing application of machine learning to model cancer outcomes in lower-middle-income regions (74). It revealed significant gaps in model development and recommended retraining models with the help of larger datasets; it also emphasised the need to enhance external validation techniques and conduct more impact assessments through randomised controlled trials.

Data is crucial for training AI systems (75). Advanced processing technologies applied to radiology report databases can enhance search and retrieval, aiding diagnostic efforts (76). In this study, we observed that research frequently utilises data from various online sources; however, the datasets are often limited in size and predominantly derived from common databases. Out of 55 studies, 26 used data from different online databases, with many sourcing data from the Kaggle repository, and others from personal medical databases, GitHub, and online libraries. Advocating for globally interconnected networks that aggregate diverse patient data is essential to optimise AI's capabilities, particularly for diseases like OPMDs and Oral Cancer, which require varied image databases. Effective curation of well-annotated medical data into large-scale databases is vital (77). However, inadequate curation remains a significant barrier to AI development (78). Proper curation—encompassing patient selection and image segmentation—ensures high-quality, error-free data and mitigates inconsistencies from varied data collection methods and imaging protocols (78, 79). Global collaborative initiatives, such as The Cancer Imaging Archive which creates extensive labelled datasets, are key to addressing this issue.

In our systematic review, 18 of the 55 studies meeting inclusion criteria provided relevant data for developing contingency tables. Metrics such as precision, F1 score, and recall, while standard in computer science, are insufficient alone for this purpose (80). Additionally, heatmaps from AI models highlight important image features for classification; they also help in the reduction of bias. However, only one-third of studies provide this information (81). Therefore, future AI-based research should prioritise establishing clear and well-defined metrics that bridge the disciplines of healthcare and computer science. In this review, we observed that the same terms are often defined inconsistently across different studies. For instance, the term “validation” is sometimes used to refer to the dataset used for evaluating model performance (82). Most research indicated that training an AI model typically involved dividing the dataset into training and testing subsets. Altman et al. recommended the use of internal validation sets for in-sample assessments and external validation sets for out-of-sample evaluations to enhance the quality of the study (83).

Histopathological imagery demonstrated superior sensitivity and specificity, while photographic images exhibited reduced accuracy. Given that the photos utilised for training deep learning models may not encompass the complete spectrum of oral disease presentations, the algorithm might encounter difficulties in consistently identifying various forms of oral lesions (84). Sub-group analysis revealed histopathological images had the highest DOR (460.83; and sensitivity 0.97), followed by OCT images (DOR 63.45; sensitivity 0.90) and photographic images (DOR 23.53; sensitivity 0.82), differing from previous reviews (85). In AI with deep learning, images are analysed to screen and detect diseases with exceptional accuracy (86) However, medical diagnostic images often reveal significant intra-class homogeneity, which complicates the extraction of nuanced features essential for precise predictions. Additionally, the relatively small size of these datasets compared to natural image datasets restricts the direct application of advanced modelling techniques. Utilising specialised knowledge and contextually relevant features can support the refinement of feature representations and alleviate model complexity, thereby advancing performance in the realm of medical diagnostic imaging (87). Most studies lacked guidelines for image data preparation before training models, Notably, Lin et al. offered a comprehensive procedure for capturing images, using a phone camera grid to ensure the lesion is centred, thus minimising focal length issues in oral photographic images (6).

Despite AI's potential in radiology, challenges persist, such as improving interpretability, reliability, and generalizability. AI's opaque decision-making limits clinical acceptance, requiring further validation through large-scale multicentre studies (88). Effective AI implementation on the one hand can reduce the unnecessary time being invested in conducting procedures, and facilitate early detection as well as improve patient outcomes on the other hand.

This study's constraints may influence both the understanding and broader application of the findings. First, the meta-analysis relies on published literature, which, despite thorough searches, may be subject to publication and language biases, especially because we included studies published only in English. Second, differences in study scale, methodological approaches, and evaluation metrics across studies have introduced inconsistencies that might influence the findings. Despite the execution of sensitivity analyses, the impact of this heterogeneity cannot be entirely discounted. Moreover, variations in imaging tools, equipment standards, and methodologies among studies could affect diagnostic accuracy.

## 5 Conclusion

This review highlights the high accuracy of AI algorithms in diagnosing oral cancer and OPMDs through medical imaging. The findings demonstrate that AI is a reliable approach for early detection, particularly in resource-limited settings. The successful integration of AI-based diagnostics, utilising various imaging modalities, highlights its potential. The widespread use of mobile devices has further expanded the accessibility of this technology, providing crucial healthcare support where specialised medical care is limited. Achieving precise image-based diagnosis with AI requires standardised methodologies and large-scale, multicentric studies. Such measures are significant for ensuring the accuracy and efficiency of screening processes and enhancing overall healthcare outcomes.

## Data Availability

The original contributions presented in the study are included in the article/Supplementary Material, further inquiries can be directed to the corresponding author.
