# Supplementary material for: Diagnostic performance of artificial intelligence in detecting oral potentially malignant disorders and oral cancer using medical diagnostic imaging: a systematic review and meta-analysis
Source: Front Oral Health. 2024 Nov 6;5:1494867. doi: 10.3389/froh.2024.1494867 (PMC11576460; doi:10.3389/froh.2024.1494867)

**Supplementary file 1. Detailed search strategies**

| **Database** | **Terms** | **Total Studies** |
| --- | --- | --- |
| **PubMed** | ("artificial intelligence*"[Mesh] OR "artificial intelligence"[tiab] OR "machine learning"[tiab] OR "deep learning"[tiab] OR "expert systems"[tiab] OR "pattern recognition"[tiab] OR AI[tiab] OR ML[tiab] OR DL[tiab] OR NLP[tiab] OR Robotics[tiab] OR "expert systems"[tiab] OR "neural networks"[tiab] OR "pattern recognition automated"[tiab] OR "data mining"[tiab] OR "decision support systems"[tiab] OR "image processing computer assisted"[tiab]) AND ("mouth neoplasms"[Mesh] OR "mouth neoplasms/diagnosis"[Mesh] OR "oral cancer"[tiab] OR "mouth mucosa"[tiab] OR "mouth cancer"[tiab] OR "oral neoplasms"[tiab] OR "mouth neoplasms"[tiab] OR "oral squamous cell carcinoma"[tiab] OR "mouth squamous cell carcinoma"[tiab] OR "oral pre cancer"[tiab] OR "oral lesions"[tiab] OR "oral mucosa cancer"[tiab]) AND ("diagnostic imaging"[Mesh] OR "medical images"[tiab] OR "radiographic images"[tiab] OR "ultrasound images"[tiab] OR "computed tomography scans"[tiab] OR "tomography images"[tiab] OR "x ray images"[tiab] OR "image processing"[tiab] OR "computer assisted image interpretation"[tiab] OR "diagnostic imaging"[tiab] OR "image based diagnosis"[tiab] OR "image guided procedures"[tiab] OR "image quality assessment"[tiab] OR "image processing computer assisted"[tiab] OR "image interpretation computer assisted"[tiab] OR "image based diagnosis"[tiab]) | 86 |
| **Scopus** | ("artificial intelligence*" OR "artificial intelligence" OR "machine learning" OR "deep learning" OR "expert systems" OR "pattern recognition" OR AI OR ML OR DL OR NLP OR Robotics OR "expert systems" OR "neural networks" OR "pattern recognition automated" OR "data mining" OR "decision support systems" OR "image processing computer assisted") AND ("mouth neoplasms" OR "mouth neoplasms/diagnosis" OR "oral cancer" OR "mouth mucosa" OR "mouth cancer" OR "oral neoplasms" OR "mouth neoplasms" OR "oral squamous cell carcinoma" OR "mouth squamous cell carcinoma" OR "oral pre cancer" OR "oral lesions" OR "oral mucosa cancer") AND ("diagnostic imaging" OR "medical images" OR "radiographic images" OR "ultrasound images" OR "computed tomography scans" OR "tomography images" OR "x ray images" OR "image processing" OR "computer assisted image interpretation" OR "diagnostic imaging" OR "image based diagnosis" OR "image guided procedures" OR "image quality assessment" OR "image processing computer assisted" OR "image interpretation computer assisted" OR "image based diagnosis") | 165 |
| **IEEE** | ("artificial intelligence*" OR "artificial intelligence" OR "machine learning" OR "deep learning" OR "expert systems" OR "pattern recognition" OR AI OR ML OR DL OR NLP OR Robotics OR "expert systems" OR "neural networks" OR "pattern recognition automated" OR "data mining" OR "decision support systems" OR "image processing computer assisted") AND ("mouth neoplasms" OR "mouth neoplasms/diagnosis" OR "oral cancer" OR "mouth mucosa" OR "mouth cancer" OR "oral neoplasms" OR "mouth neoplasms" OR "oral squamous cell carcinoma" OR "mouth squamous cell carcinoma" OR "oral pre cancer" OR "oral lesions" OR "oral mucosa cancer") AND ("diagnostic imaging" OR "medical images" OR "radiographic images" OR "ultrasound images" OR "computed tomography scans" OR "tomography images" OR "x ray images" OR "image processing" OR "computer assisted image interpretation" OR "diagnostic imaging" OR "image based diagnosis" OR "image guided procedures" OR "image quality assessment" OR "image processing computer assisted" OR "image interpretation computer assisted" OR "image based diagnosis") | 45 |

**Supplementary file 2. Quality assessment of the studies (Risk of bias and concern of applicability for each item in included studies**

| **Study** | **RISK OF BIAS** | | | | **APPLICABILITY CONCERNS** | | |
| --- | --- | --- | --- | --- | --- | --- | --- |
|  | **PATIENT SELECTION** | **INDEX TEST** | **REFERENCE STANDARD** | **FLOW AND TIMING** | **PATIENT SELECTION** | **INDEX TEST** | **REFERENCE STANDARD** |
| Al Duhayyim et al, 2023 | ☺ | ☹ | ? | ☺ | ☺ | ? | ? |
| Alanazi et al, 2022 | ☺ | ☹ | ? | ☺ | ☺ | ? | ? |
| Bansal et al, 2023 | ☺ | ☹ | ? | ☺ | ? | ☺ | ? |
| Deif et al, 2022 | ☺ | ? | ☺ | ? | ☺ | ☺ | ☺ |
| Fati SM et al, 2022 | ☺ | ☹ | ☺ | ☺ | ☺ | ☺ | ☺ |
| Gupta et al, 2020 | ☺ | ☹ | ☺ | ? | ☺ | ☺ | ☺ |
| James et al, 2021 | ☺ | ☹ | ☺ | ☺ | ☺ | ☺ | ☺ |
| Lin et al, 2021 | ☺ | ☹ | ☺ | ☺ | ☺ | ☺ | ☺ |
| Marzouk et al, 2022 | ☺ | ☹ | ? | ☺ | ☺ | ? | ? |
| Yuan W et al, 2022 | ☺ | ☹ | ☺ | ☺ | ☺ | ☺ | ☺ |
| Warin K et al, 2022 | ☺ | ☹ | ☺ | ? | ☺ | ☺ | ☺ |
| Panigrahi S et al, 2023 | ☺ | ☹ | ☺ | ☺ | ☺ | ? | ☺ |
| Yang SY et al, 2022 | ☺ | ☹ | ☺ | ☺ | ☺ | ☺ | ☺ |
| Muqeet MA et al, 2022 | ☺ | ☹ | ? | ☺ | ? | ☺ | ☺ |
| Welikala RA et al, 2020 | ☺ | ☹ | ☺ | ☺ | ☺ | ☺ | ☺ |
| Goswami M et al, 2021 | ☺ | ☹ | ☺ | ☺ | ☺ | ☺ | ☺ |
| Xue Z et al, 2022 | ☺ | ☹ | ☺ | ☺ | ☺ | ☺ | ☺ |
| Huang et al, 2022 | ☺ | ☹ | ☺ | ☺ | ☺ | ☺ | ☺ |

☺Low Risk ☹High Risk ? Unclear Risk

**QUADAS-AI summary plot**

**
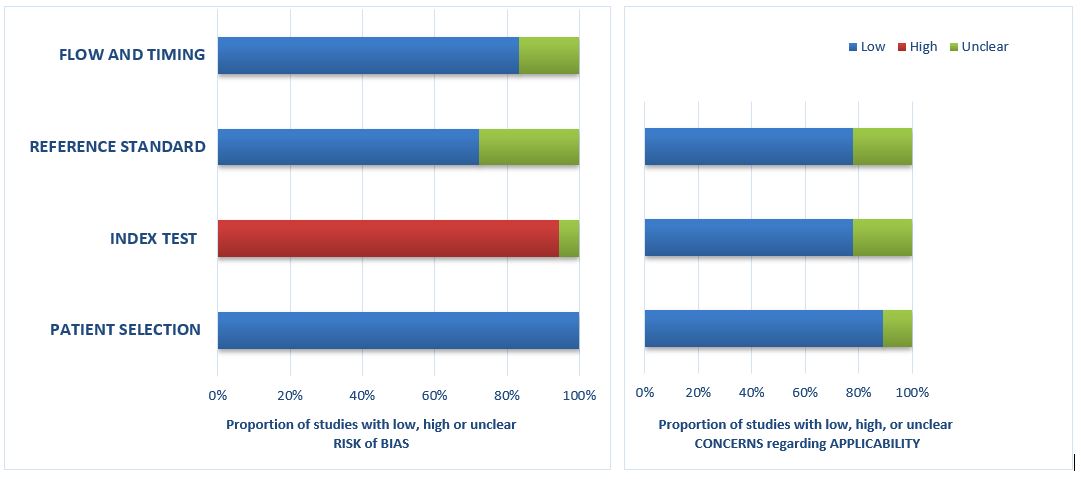
**

**Supplementary file 3. Overall Diagnostic performance of AI models with medical images for detecting OPMDs & Oral cancer**


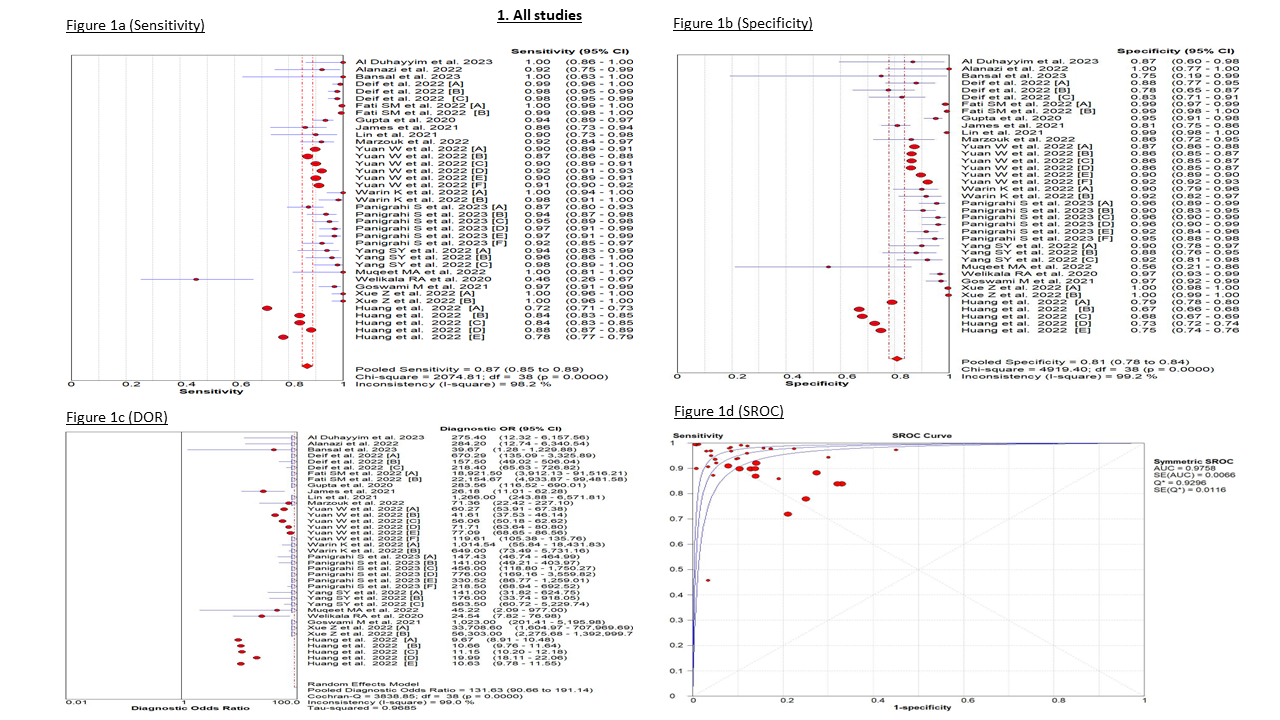


**Supplementary file 4. Diagnostic Performance of various AI models for Detecting OPMDs & Oral Cancer**


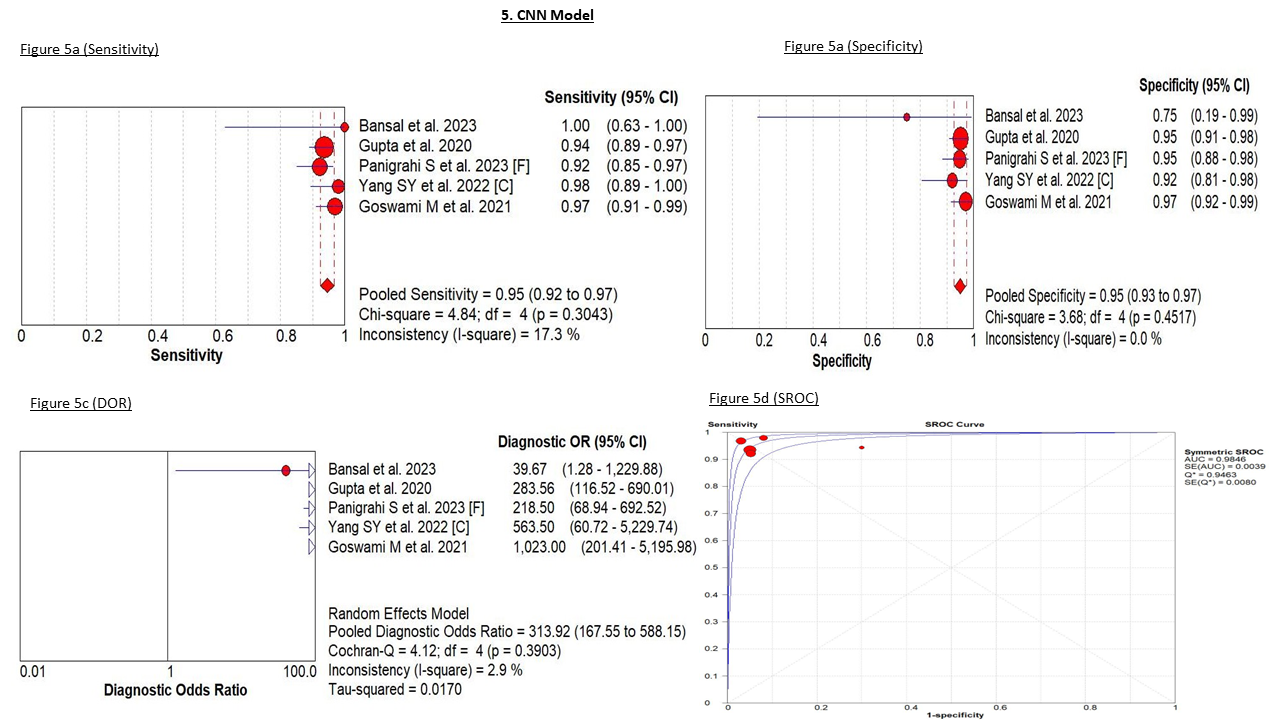


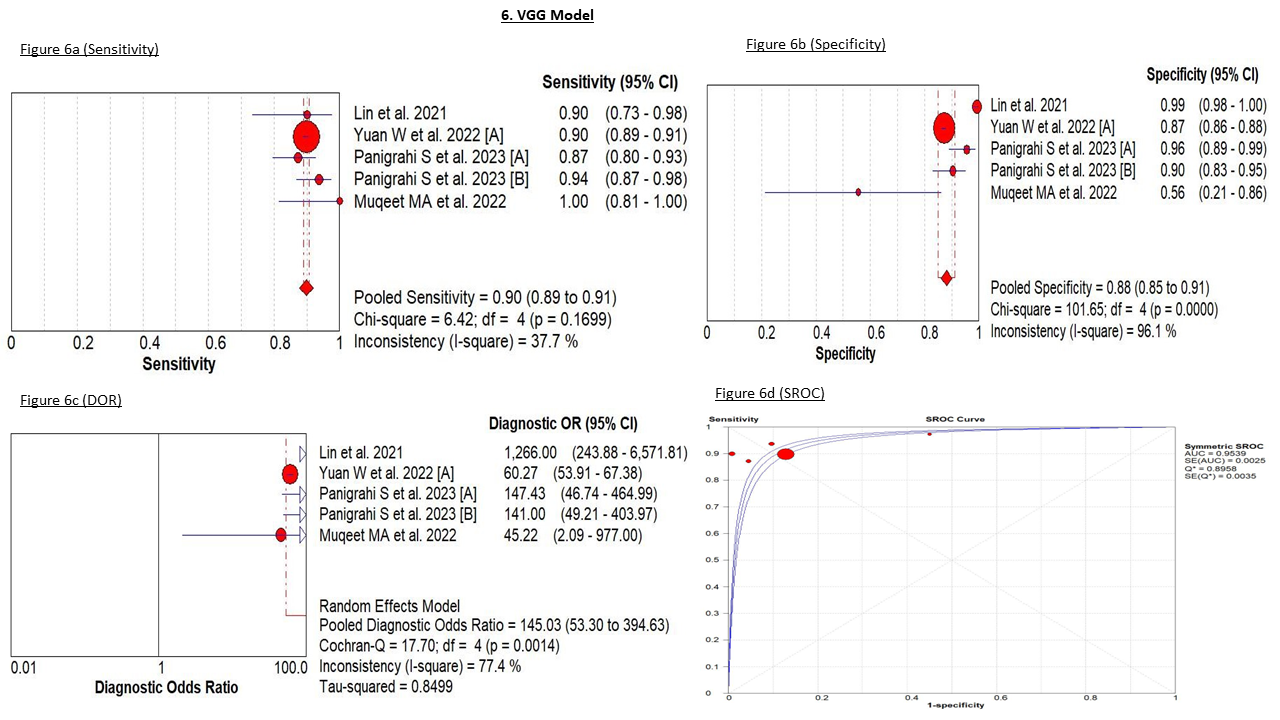


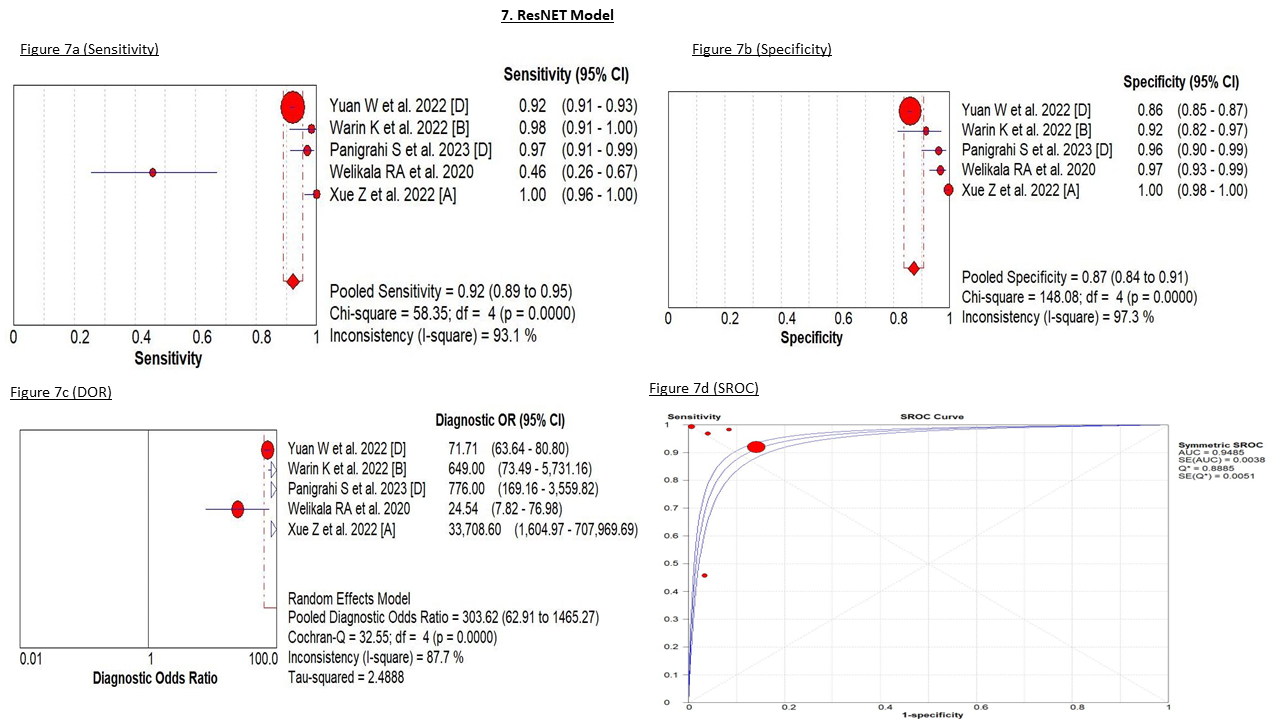


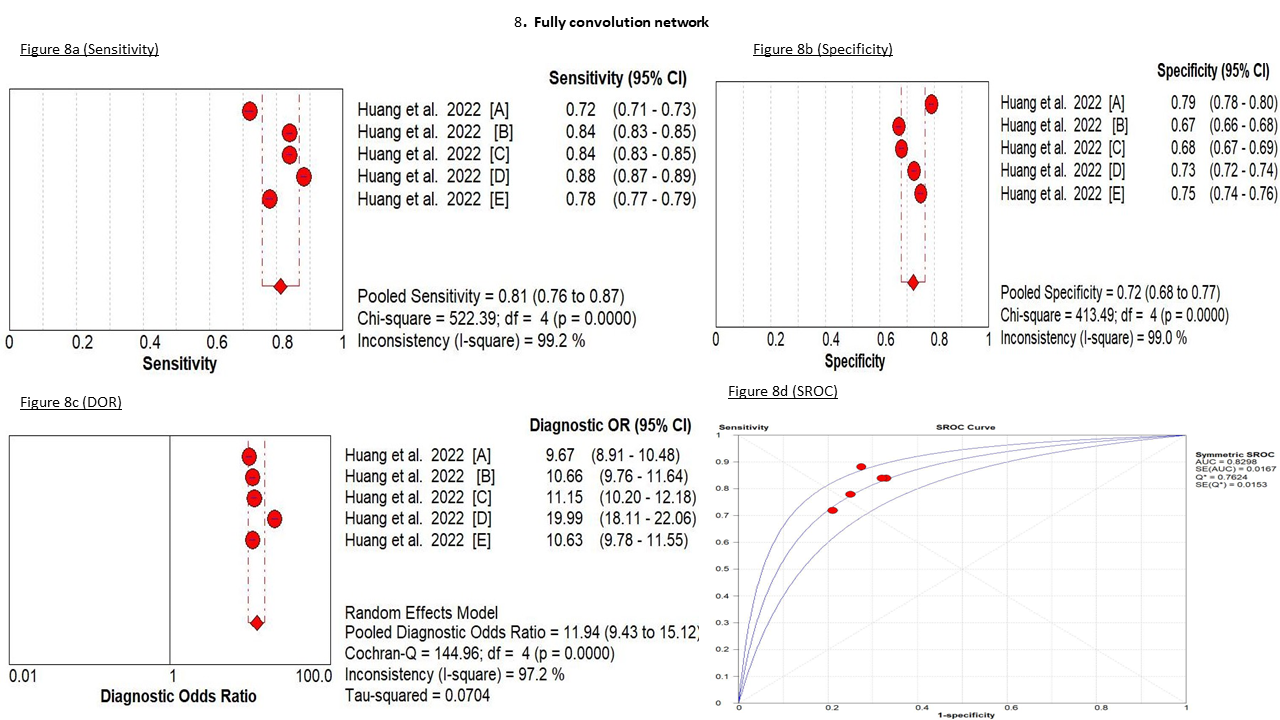


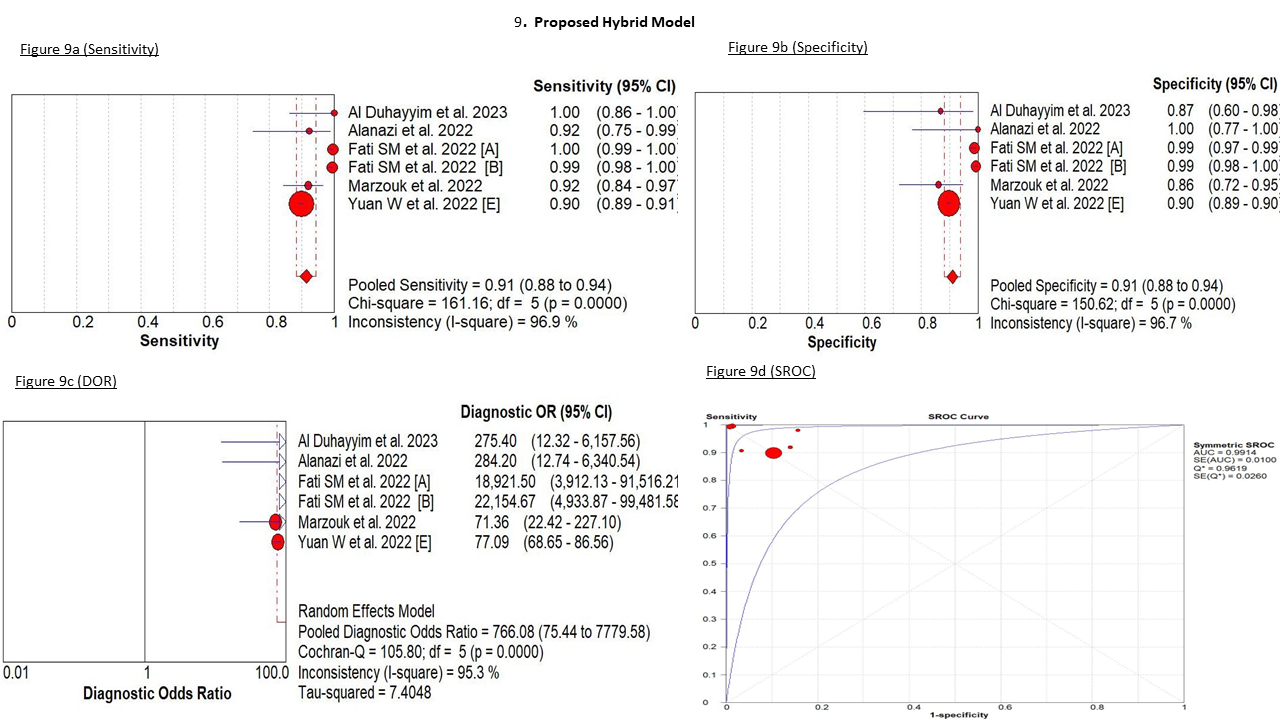


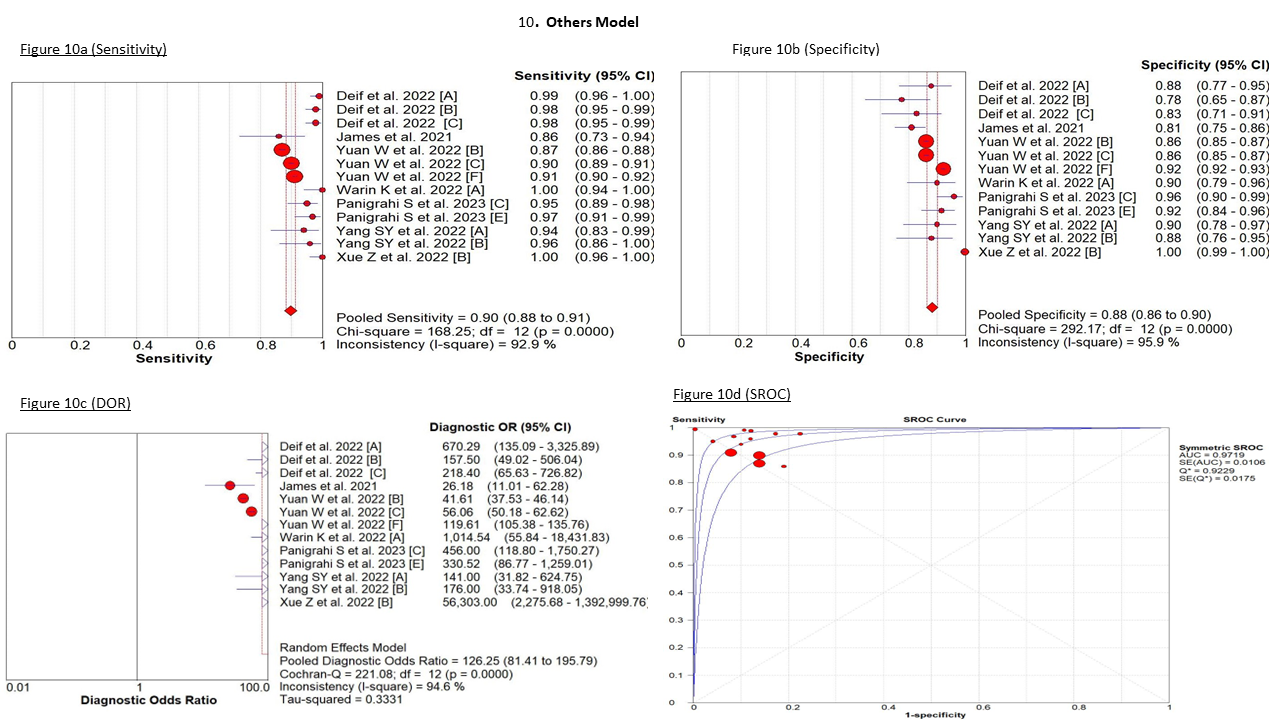


**Supplementary file 5. Image-Based Diagnostic Performance of AI Models for Detecting OPMDs & Oral Cancer**
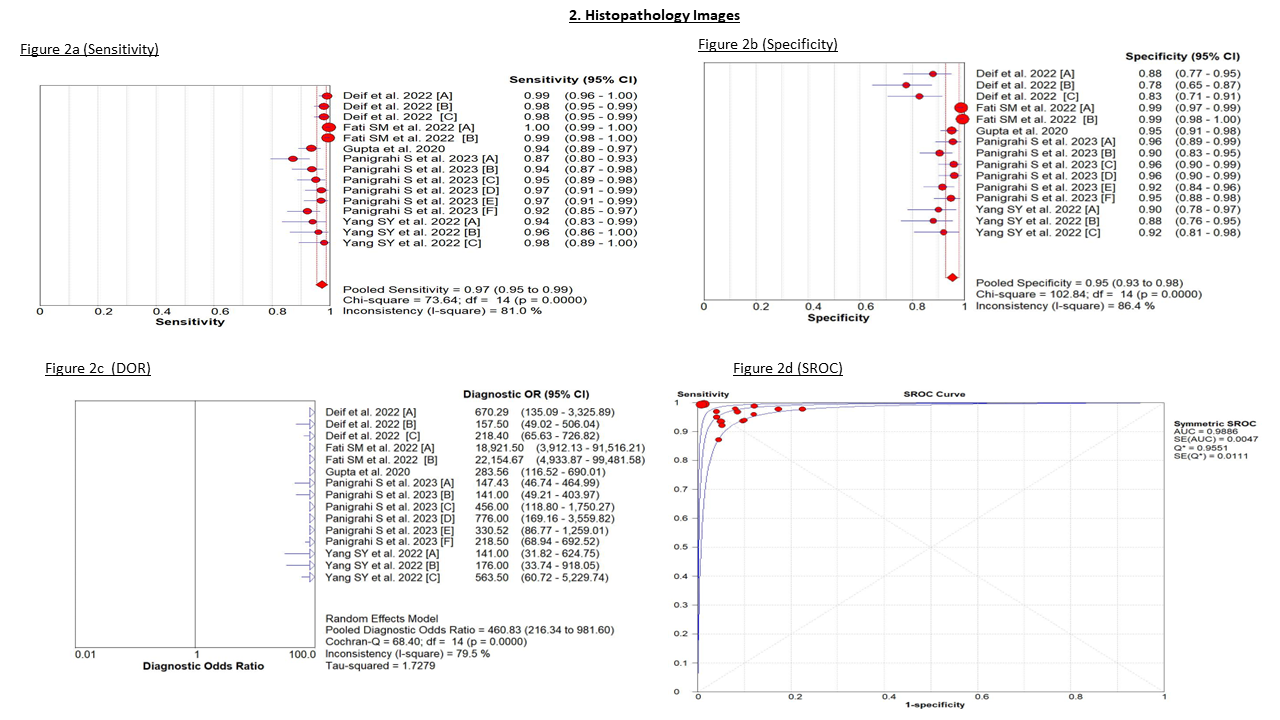


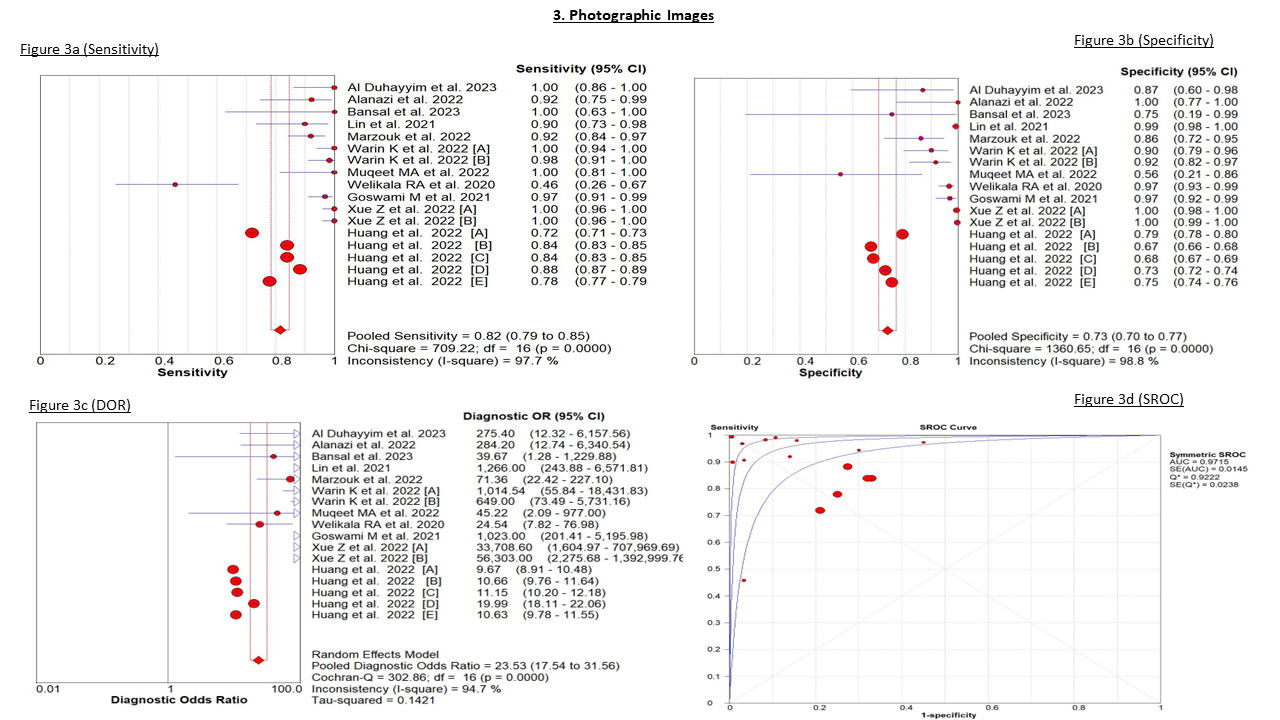


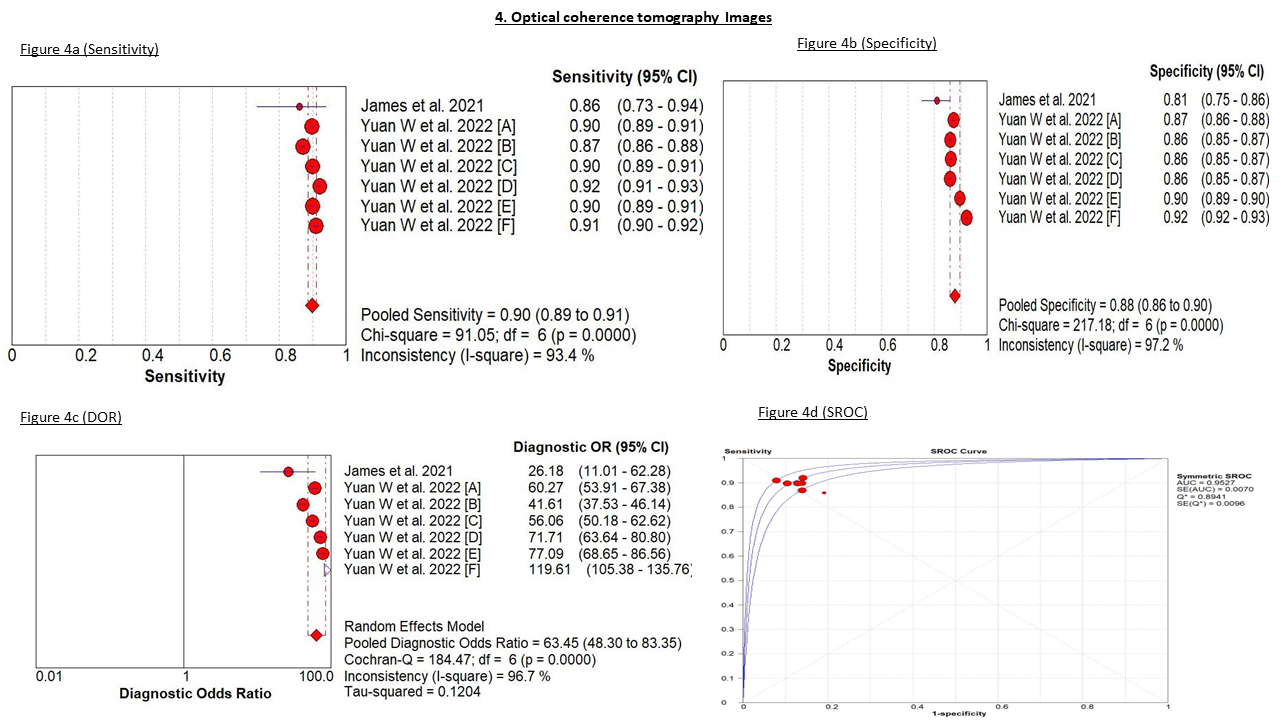


**Supplementary file 6. Diagnostic Performance of AI Models for Detecting Across Oral Conditions (OPMDs Oral Cancer & both)**


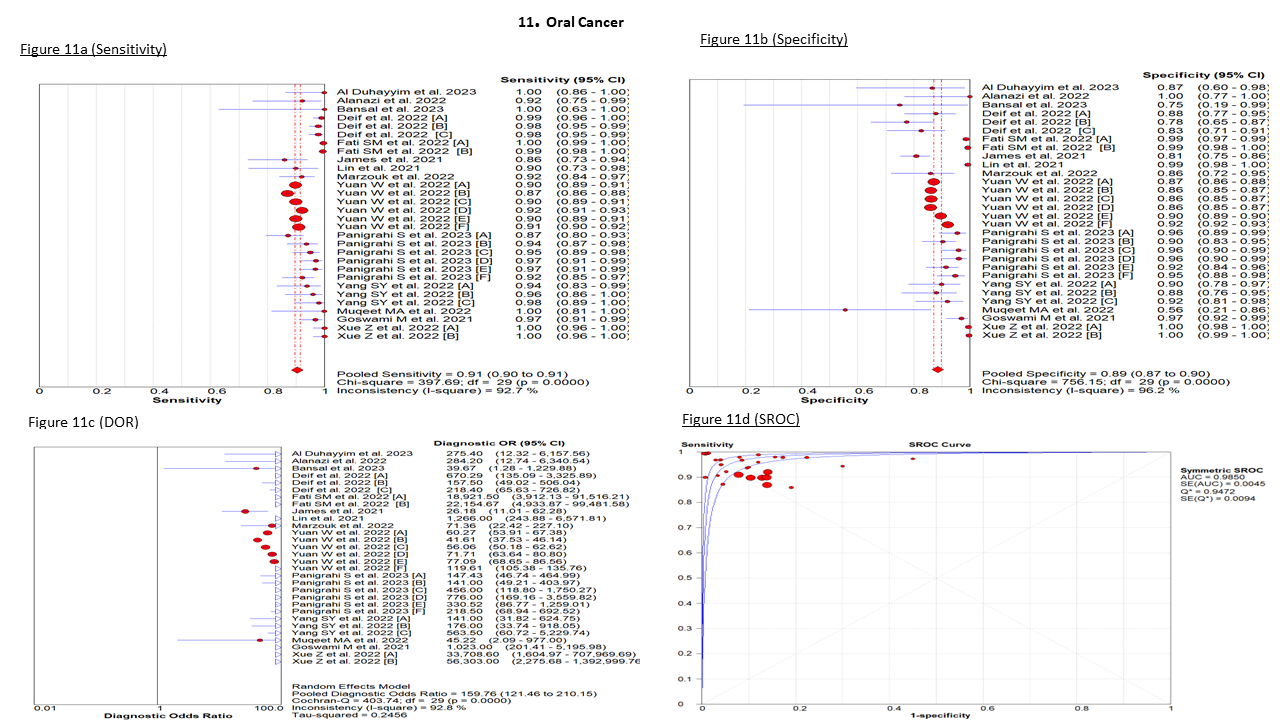


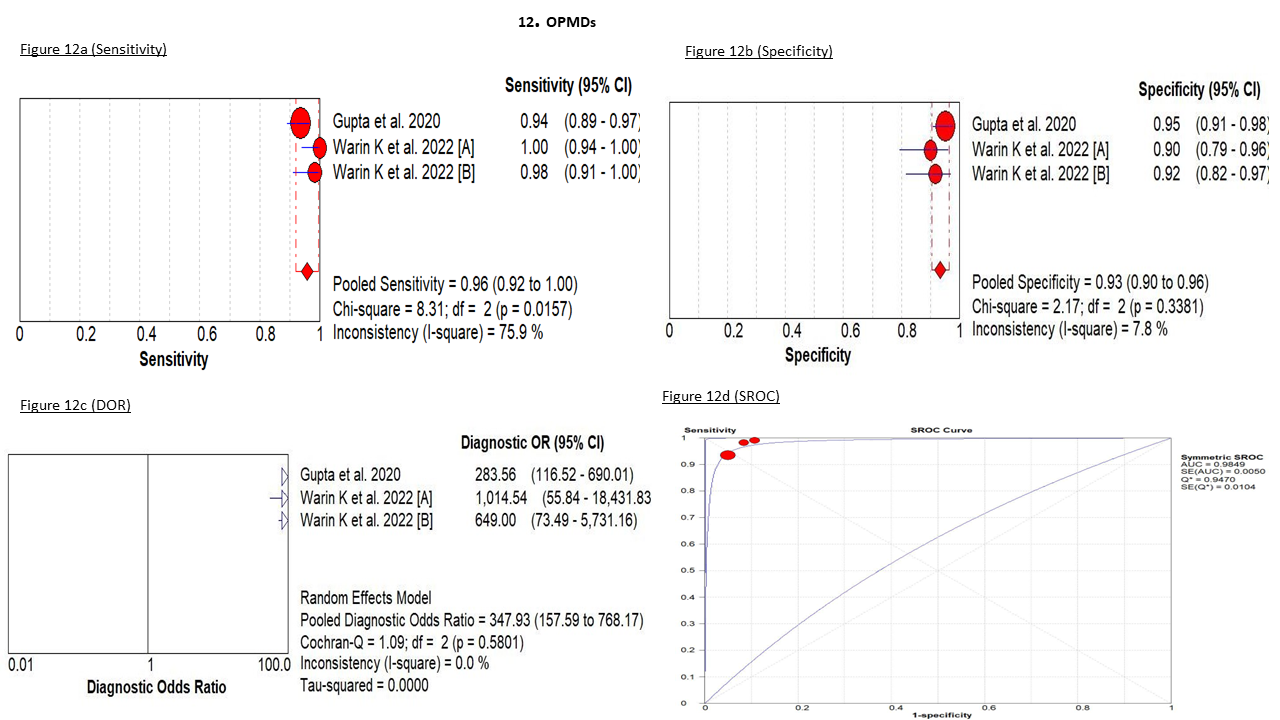


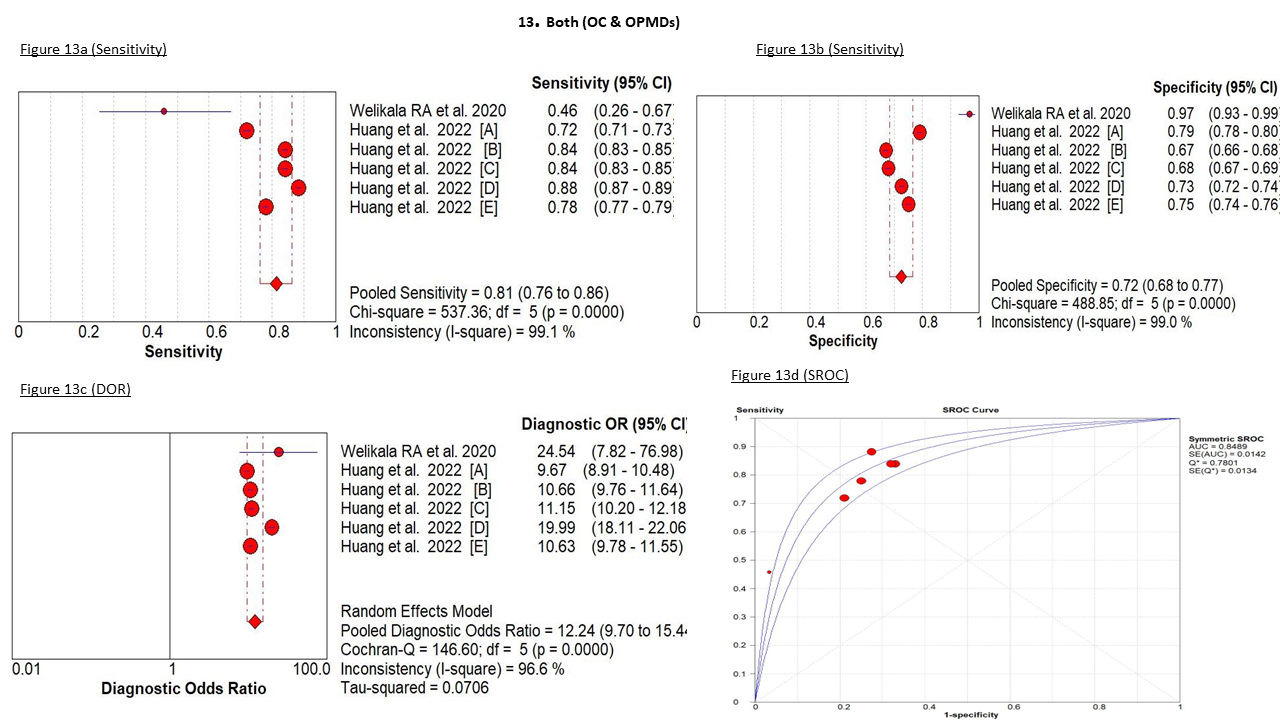


**Supplementary file 7. Diagnostic performance of AI models for detecting OPMDs & Oral cancer across different Income groups**


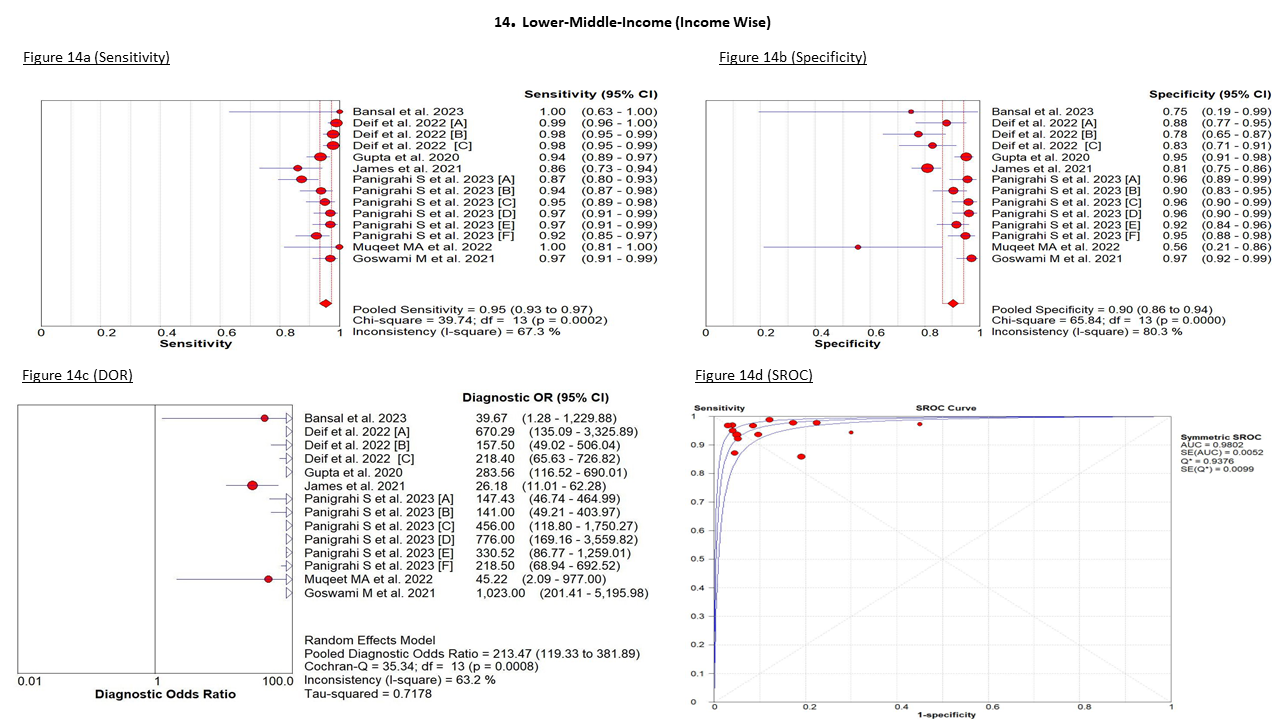


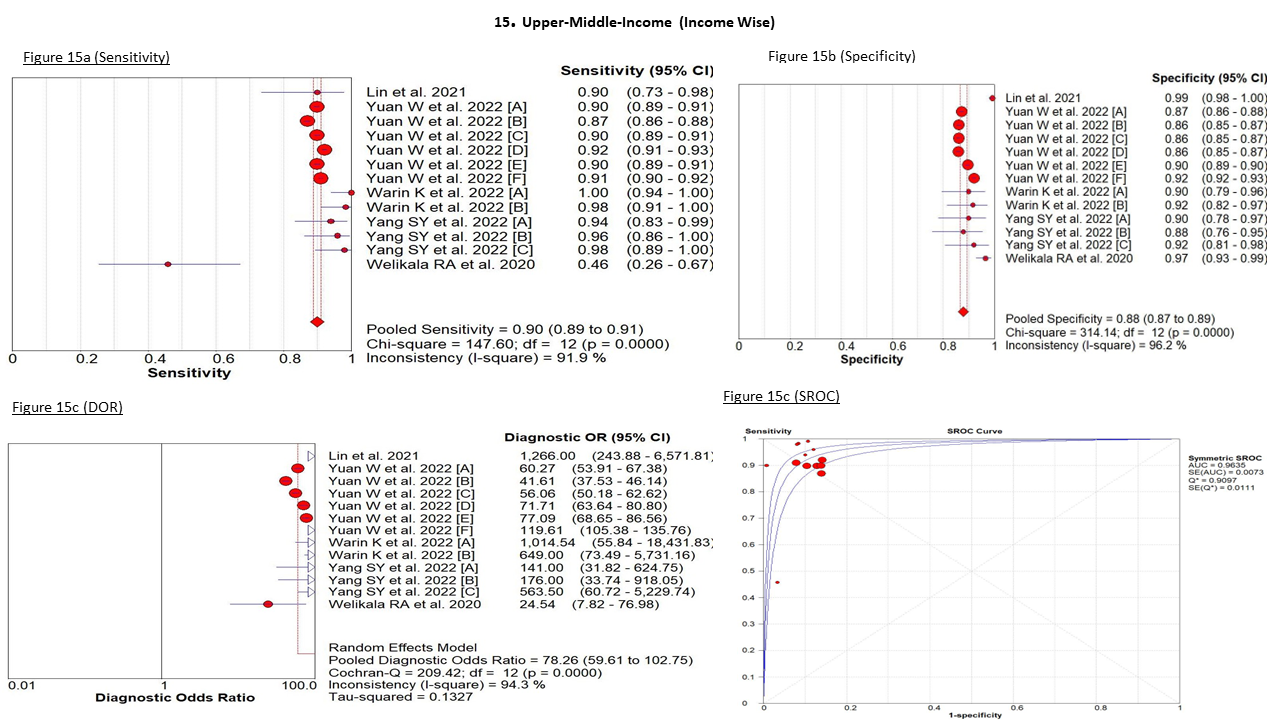


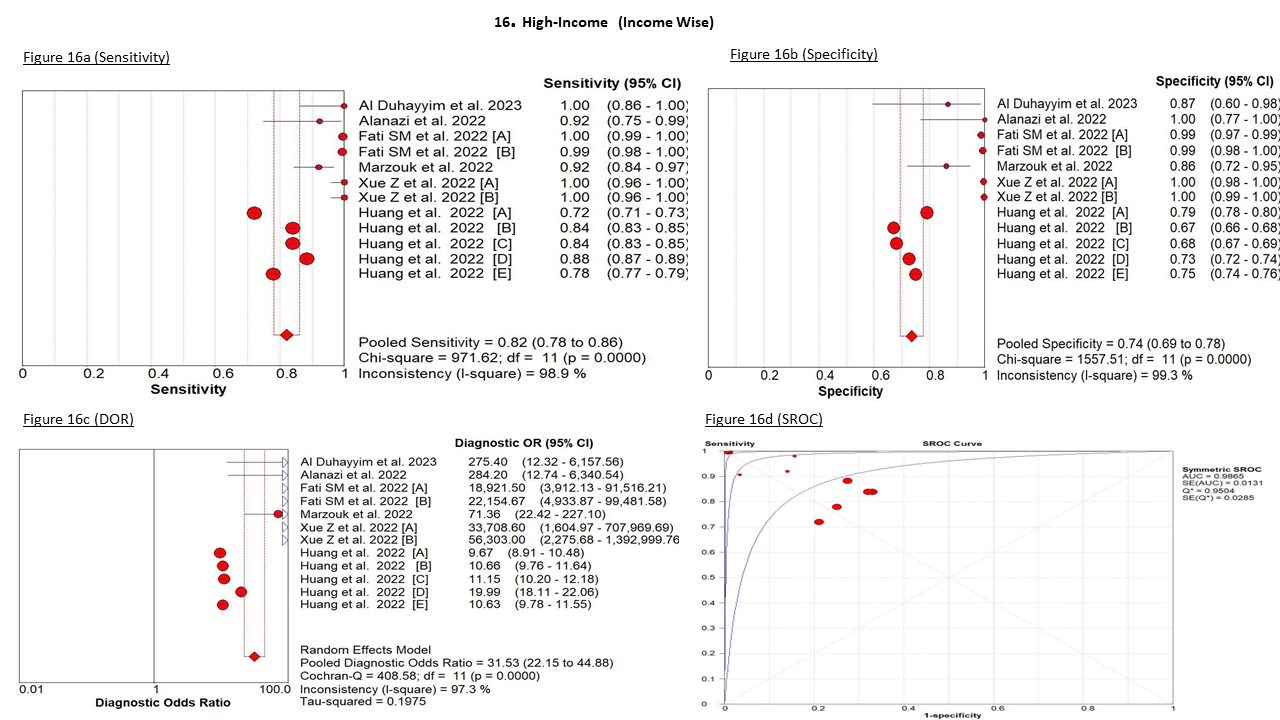


**Supplementary file 8. Region-Based Diagnostic Performance of AI Models for Detecting OPMDs and Oral Cancer**


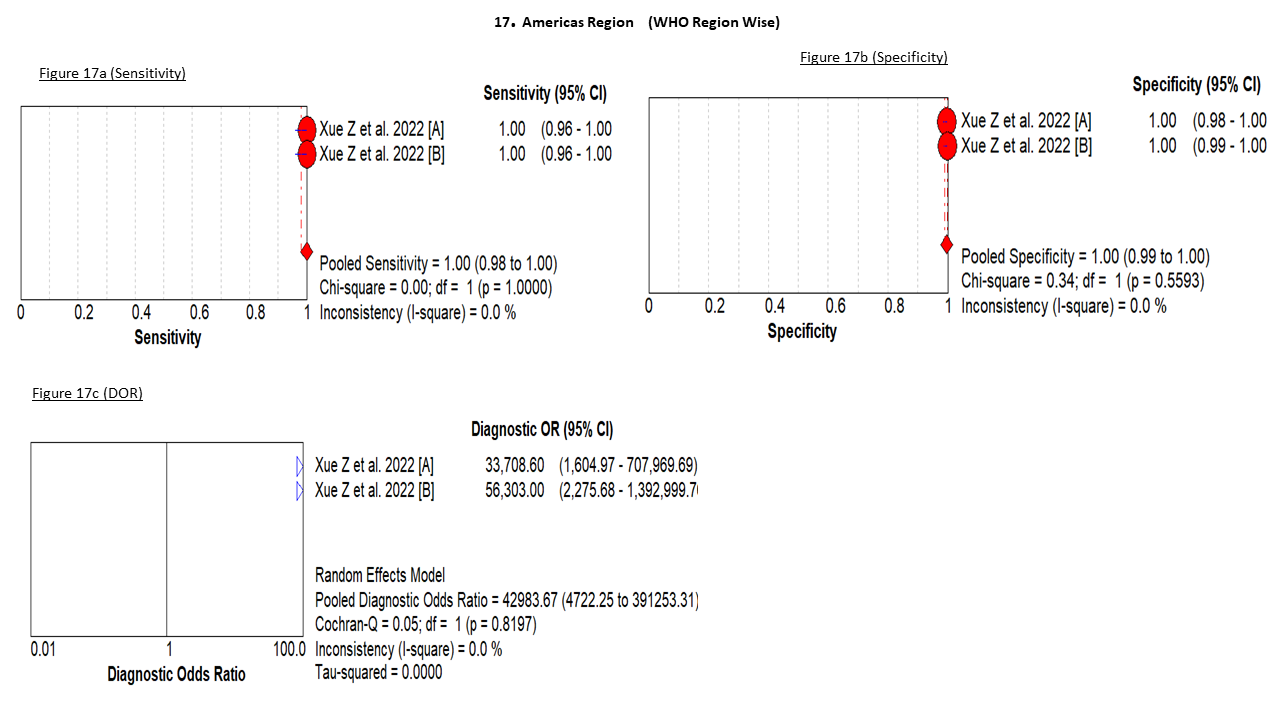


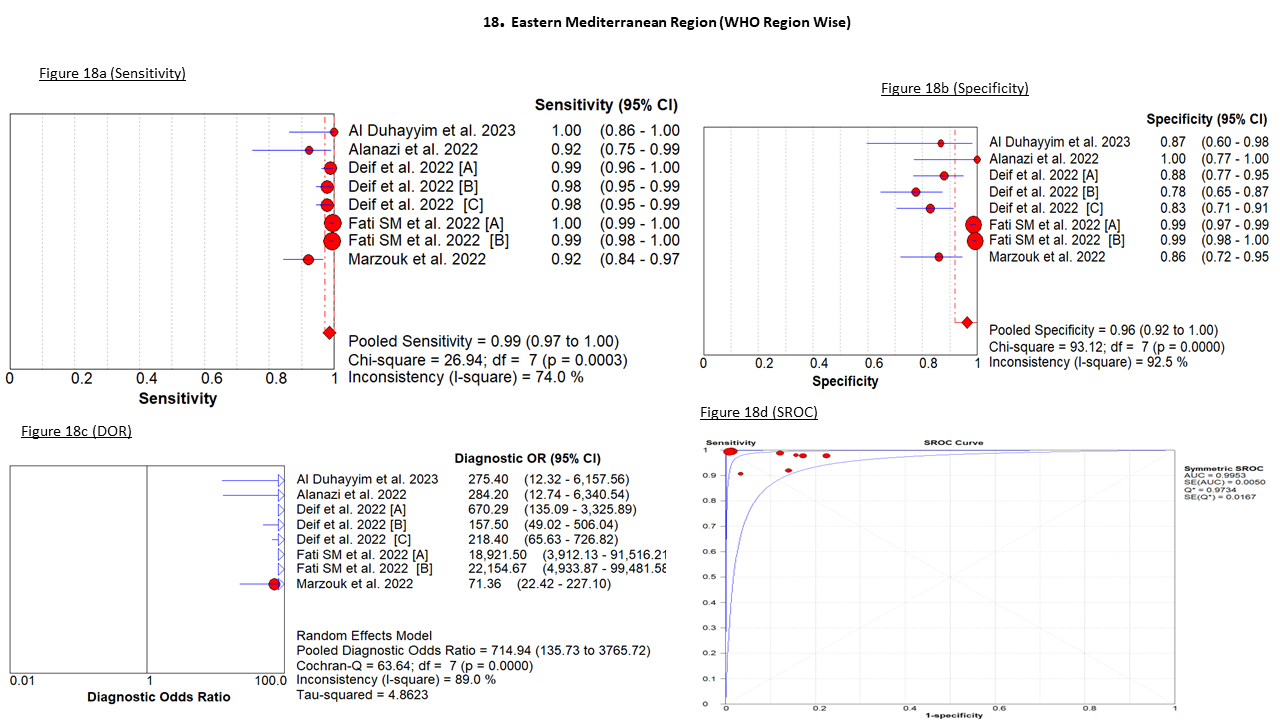


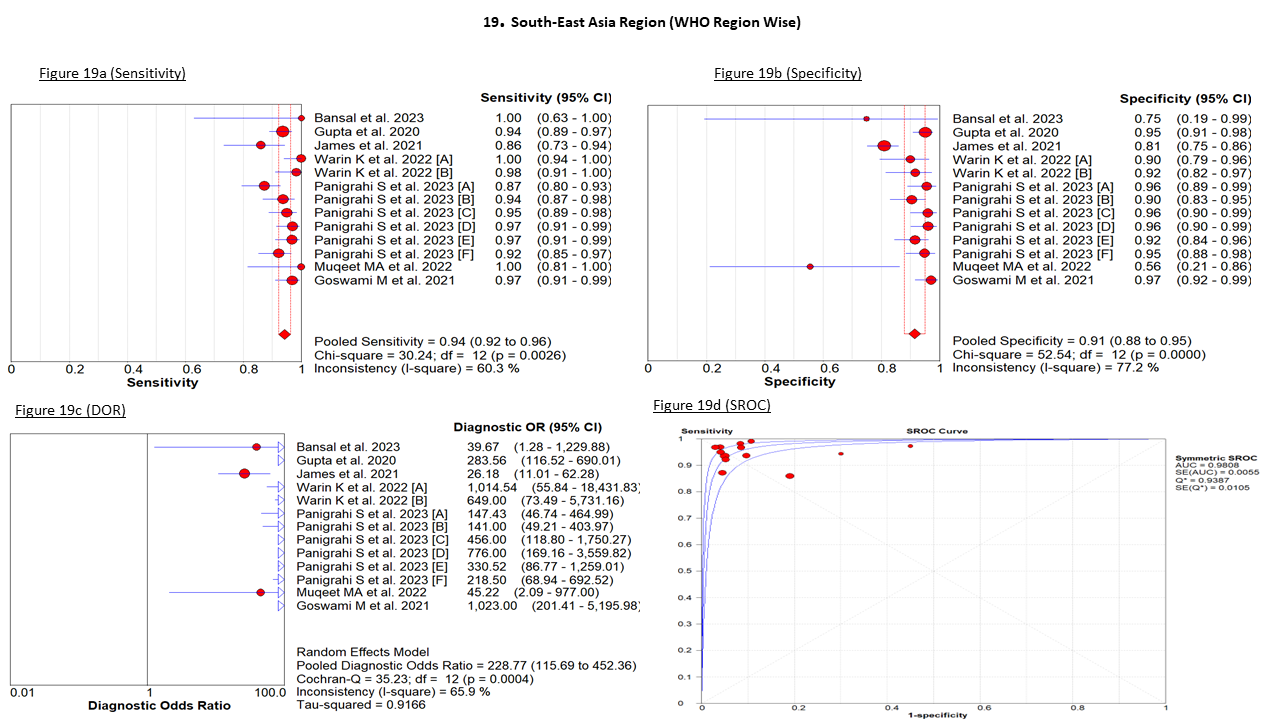


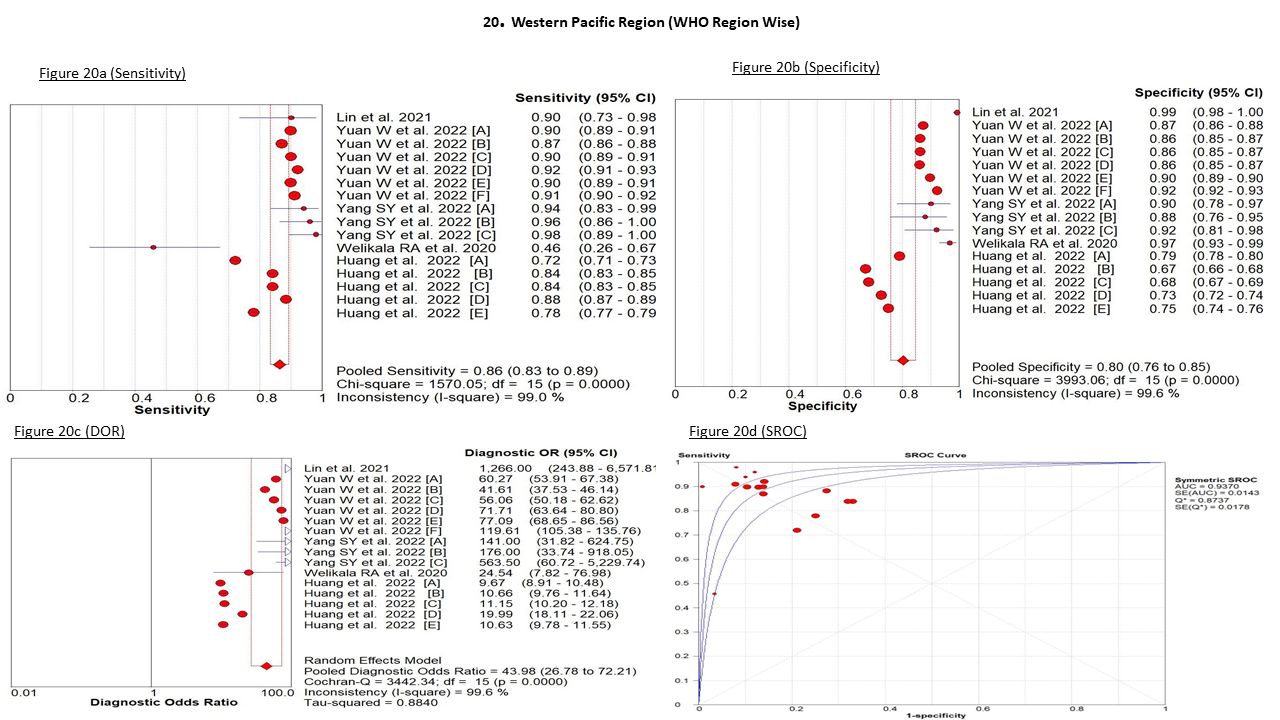

Supplement: Supplementary file 1 [file Table1.docx]
